# Supplementary material for: Novel Triazeneindole Antibiotics: Synthesis and Hit-to-Lead Optimization
Source: Int J Mol Sci. 2025 Feb 21;26(5):1870. doi: 10.3390/ijms26051870 (PMC11899342; doi:10.3390/ijms26051870)
Supplement: Supplementary file 1 [file ijms-26-01870-s001.zip › Supplementary File and Figures Captions.docx]

**Supplementary Figure S1.** UPLC-MS/MS data for compound BX: (**A**) mass-spectrum, (**B**) MRM chromatogram.

**Supplementary Figure S2.** Stability of BX-SI043 and chlorambucil used as a control in gastric and intestinal juices.

**Supplementary Figure S3.** Stability of BX-SI043 and verapamil used as a control in human and rat liver microsomes.

**Supplementary Figure S4**. Stability of BX-SI043 and eucatropine used as a control in human and rat plasma.

**Supplementary Figure S5.** Inhibition of hERG ion channels by BX-SI043 and E-4031 used as a control.

**Supplementary Table S1.** chemical structures and drug-likeness parameters of initial virtual library.

**Supplementary Table S2.** Yield and solubility of synthesized compounds in water.

**Supplementary Table S3.** Drug sensibility profile of MRSA clinical isolates.

**Supplementary Table S4.** MIC of BX-SI043 vs BX-SI001 on 51 clinical isolate.

**Supplementary Table S5.** Stability of ВХ-SI043 and verapamil used as a control in the microsomal human and rat liver microsomes.

**Supplementary Table S6.** Inhibition of the cytochrome activity in the microsomal liver fraction by BX-SI043 and corresponding isoform inhibitors.

**Supplementary Table S7.** Permeability and asymmetry of transport of test and control compounds in the Caco-2 cell model.

**Supplementary Table S8.** Effect of the test substance on body weights of experimental animals at intragastric administration.

**Supplementary Table S9.** Effect of the test substance on relative organ weight of experimental rats after 15 days of the study, % of body weight, (M±SEM).

**Supplementary File S1.** ^1^Н and ^13^С NMR spectra of synthesized compounds.
