# Supplementary material for: Novel Triazeneindole Antibiotics: Synthesis and Hit-to-Lead Optimization
Source: Int J Mol Sci. 2025 Feb 21;26(5):1870. doi: 10.3390/ijms26051870 (PMC11899342; doi:10.3390/ijms26051870)
Supplement: Supplementary file 1 [file ijms-26-01870-s001.zip › Supplementary Tables S1-S9.docx]

**Supplementary Table S1.** Chemical structures and drug-likeness parameters of initial virtual library.

| Compound | Structure SMILES | Molar mass | LogP | TPSA | nON | nOHNH | nRotB |
| --- | --- | --- | --- | --- | --- | --- | --- |
| BX- SI001 | CCOC(=O)c2[nH]c1ccccc1c2N=NN3CCN(C)CC3 | 315.38 | 3,19 | 73.3 | 7 | 1 | 5 |
| BX-SI002 | CCOC(=O)c2[nH]c1ccccc1c2N=NN(CC)CC | 288.35 | 4.05 | 70.6 | 6 | 1 | 7 |
| BX-SI003 | CCOC(=O)c2[nH]c1ccccc1c2N=NN(C)Cc3ccccc3 | 336.39 | 4.7 | 70.06 | 6 | 1 | 7 |
| BX-SI004 | CCOC(=O)c2[nH]c1ccccc1c2N=NN4CCN(Cc3ccccc3)CC4 | 391.48 | 4.59 | 73.7 | 7 | 1 | 7 |
| BX-SI005 | CCOC(=O)c2[nH]c1ccccc1c2N=NN3CCCN(C)CC3 | 329.4 | 3.46 | 73.3 | 7 | 1 | 5 |
| BX-SI006 | CCOC(=O)c2[nH]c1ccccc1c2N=NN3CCOCC3 | 302.33 | 3.14 | 79.3 | 7 | 1 | 5 |
| BX-SI007 | CCOC(=O)c2[nH]c1ccccc1c2N=NN3CCC(C(=O)OCC)CC3 | 372.43 | 4.31 | 96.37 | 8 | 1 | 8 |
| BX-SI008 | CCOC(=O)c2[nH]c1ccccc1c2N=NN3CCC(O)CC3 | 316.36 | 2.32 | 90.29 | 7 | 2 | 5 |
| BX-SI009 | CCOC(=O)c2[nH]c1ccccc1c2N=NN3CCCC3 | 286.33 | 3.7 | 70.06 | 6 | 1 | 5 |
| BX-SI010 | CCOC(=O)c2[nH]c1ccccc1c2N=NN(C)CCN(C)C | 317.39 | 3.33 | 73.3 | 7 | 1 | 8 |
| BX-SI011 | CCOC(=O)c2[nH]c1ccccc1c2N=NN3CCC(C)CC3 | 314.39 | 4.45 | 70.06 | 6 | 1 | 5 |
| BX-SI012 | CCOC(=O)c2[nH]c1ccccc1c2N=NN(C)CC3CCOCC3 | 344.42 | 4.05 | 79.3 | 7 | 1 | 7 |
| BX-SI013 | CCOC(=O)c2[nH]c1ccccc1c2N=NN3CCC(CCO)CC3 | 344.42 | 3.89 | 90.29 | 7 | 2 | 7 |
| BX-SI014 | CCOC(=O)c2[nH]c1ccccc1c2N=NN(C)CCc3ccccn3 | 351.41 | 3.93 | 82.95 | 7 | 1 | 8 |
| BX-SI015 | CCOC(=O)c2[nH]c1ccccc1c2N=NN4CCc3ccccc3C4 | 348.41 | 4.69 | 70.06 | 6 | 1 | 5 |
| BX-SI016 | CCOC(=O)c2[nH]c1ccccc1c2N=NN3CCC(CCOC)CC3 | 358.44 | 4.51 | 79.3 | 7 | 1 | 8 |
| BX-SI017 | CCOC(=O)c2[nH]c1ccccc1c2N=NN4CCC(c3ccc(F)cc3)CC | 394.45 | 5.93 | 70.06 | 6 | 1 | 6 |
| BX-SI018 | CCOC(=O)c3c(N=NN1CCN(C)CC1)c2cccnc2n3C | 330.39 | 2.36 | 75.33 | 8 | 0 | 5 |
| BX-SI019 | CCOC(=O)c2c(N=NN(CC)CC)c1cccnc1n2C | 303.37 | 3.22 | 72.1 | 7 | 0 | 7 |
| BX-SI020 | CCOC(=O)c3c(N=NN(C)Cc1ccccc1)c2cccnc2n3C | 351.41 | 3.87 | 72.1 | 7 | 0 | 7 |
| BX-SI021 | CCOC(=O)c4c(N=NN2CCN(Cc1ccccc1)CC2)c3cccnc3n4C | 406.49 | 3.76 | 75.33 | 8 | 0 | 7 |
| BX-SI022 | CCOC(=O)c3c(N=NN1CCCN(C)CC1)c2cccnc2n3C | 344.42 | 2.63 | 75.33 | 8 | 0 | 5 |
| BX-SI023 | CCOC(=O)c3c(N=NN1CCOCC1)c2cccnc2n3C | 317.35 | 2.31 | 81.33 | 8 | 0 | 5 |
| BX-SI024 | CCOC(=O)c3c(N=NN1CCC(C(=O)OCC)CC1)c2cccnc2n3C | 387.44 | 3.48 | 98.4 | 9 | 0 | 8 |
| BX-SI025 | CCOC(=O)c3c(N=NN1CCC(O)CC1)c2cccnc2n3C | 331.38 | 1.49 | 92.33 | 8 | 1 | 5 |
| BX-SI026 | CCOC(=O)c3c(N=NN1CCCC1)c2cccnc2n3C | 301.35 | 2.87 | 72.1 | 7 | 0 | 5 |
| BX-SI027 | CCOC(=O)c2c(N=NN(C)CCN(C)C)c1cccnc1n2C | 332.41 | 2.5 | 75.33 | 8 | 0 | 8 |
| BX-SI028 | CCOC(=O)c3c(N=NN1CCC(C)CC1)c2cccnc2n3C | 329.4 | 3.62 | 72.1 | 7 | 0 | 5 |
| BX-SI029 | CCOC(=O)c3c(N=NN(C)CC1CCOCC1)c2cccnc2n3C | 359.43 | 3.22 | 81.33 | 8 | 0 | 7 |
| BX-SI030 | CCOC(=O)c3c(N=NN1CCC(CCO)CC1)c2cccnc2n3C | 359.43 | 3.06 | 92.33 | 8 | 1 | 7 |
| BX-SI031 | CCOC(=O)c3c(N=NN(C)CCc1ccccn1)c2cccnc2n3C | 366.43 | 3.1 | 84.99 | 8 | 0 | 8 |
| BX-SI032 | CCOC(=O)c4c(N=NN2CCc1ccccc1C2)c3cccnc3n4C | 363.42 | 3.86 | 72.1 | 7 | 0 | 5 |
| BX-SI033 | CCOC(=O)c3c(N=NN1CCC(CCOC)CC1)c2cccnc2n3C | 373.46 | 3.68 | 81.33 | 8 | 0 | 8 |
| BX-SI034 | CCOC(=O)c4c(N=NN2CCN(c1ccc(F)cc1)CC2)c3cccnc3n4C | 410.45 | 4.22 | 75.33 | 8 | 0 | 6 |
| BX-SI035 | CCOC(=O)c2[nH]c1cc(F)ccc1c2N=NN3CCN(C)CC3 | 333.37 | 3.33 | 73.3 | 7 | 1 | 5 |
| BX-SI036 | CCOC(=O)c2[nH]c1cc(F)ccc1c2N=NN(CC)CC | 306.34 | 4.19 | 70.06 | 6 | 1 | 7 |
| BX-SI037 | CCOC(=O)c2[nH]c1cc(F)ccc1c2N=NN(C)Cc3ccccc3 | 354.38 | 4.84 | 70.06 | 6 | 1 | 7 |
| BX-SI038 | CCOC(=O)c2[nH]c1cc(F)ccc1c2N=NN4CCN(Cc3ccccc3)CC4 | 409.46 | 4.73 | 73.3 | 7 | 1 | 7 |
| BX-SI039 | CCOC(=O)c2[nH]c1cc(F)ccc1c2N=NN3CCCN(C)CC3 | 347.39 | 3.6 | 73.3 | 7 | 1 | 5 |
| BX-SI040 | CCOC(=O)c2[nH]c1cc(F)ccc1c2N=NN3CCOCC3 | 320.32 | 3.28 | 79.3 | 7 | 1 | 5 |
| BX-SI041 | CCOC(=O)c2[nH]c1cc(F)ccc1c2N=NN3CCC(C(=O)OCC)CC3 | 390.42 | 4.45 | 96.37 | 8 | 1 | 8 |
| BX-SI042 | CCOC(=O)c2[nH]c1cc(F)ccc1c2N=NN3CCC(O)CC3 | 334.35 | 2.46 | 90.29 | 7 | 2 | 5 |
| BX-SI043 | CCOC(=O)c2[nH]c1cc(F)ccc1c2N=NN3CCCC3 | 304.32 | 3.84 | 70.06 | 6 | 1 | 5 |
| BX-SI044 | CCOC(=O)c2[nH]c1cc(F)ccc1c2N=NN(C)CCN(C)C | 335.38 | 3.47 | 73.3 | 7 | 1 | 8 |
| BX-SI045 | CCOC(=O)c2[nH]c1cc(F)ccc1c2N=NN3CCC(C)CC3 | 332.38 | 4.59 | 70.06 | 6 | 1 | 5 |
| BX-SI046 | CCOC(=O)c2[nH]c1cc(F)ccc1c2N=NN(C)CC3CCOCC3 | 362.4 | 4.2 | 79.3 | 7 | 1 | 7 |
| BX-SI047 | CCOC(=O)c2[nH]c1cc(F)ccc1c2N=NN3CCN(CCO)CC3 | 363.39 | 2.7 | 93.53 | 8 | 2 | 7 |
| BX-SI048 | CCOC(=O)c2[nH]c1cc(F)ccc1c2N=NN(C)CCc3ccccn3 | 369.4 | 4.07 | 82.95 | 7 | 1 | 8 |
| BX-SI049 | CCOC(=O)c2[nH]c1cc(F)ccc1c2N=NN4CCc3ccccc3C4 | 366.4 | 4.83 | 70.06 | 6 | 1 | 5 |
| BX-SI050 | CCOC(=O)c2[nH]c1cc(F)ccc1c2N=NN3CCC(CCOC)CC3 | 376.43 | 4.65 | 79.3 | 7 | 1 | 8 |
| BX-SI051 | CCOC(=O)c2[nH]c1cc(F)ccc1c2N=NN4CCN(c3ccc(F)cc3)CC4 | 413.43 | 5.19 | 73.3 | 7 | 1 | 6 |
| BX-SI052 | CCOC(=O)c2[nH]c1ccccc1c2N=NN3CCCCC3 | 300.36 | 4.21 | 70.06 | 6 | 1 | 5 |
| BX-SI053 | CCOC(=O)c3c(N=NN1CCCCC1)c2cccnc2n3C | 315.38 | 3.37 | 72.1 | 7 | 0 | 5 |
| BX-SI054 | CCOC(=O)c2[nH]c1cc(F)ccc1c2N=NN3CCCCC3 | 318.35 | 4.34 | 70.06 | 6 | 1 | 5 |
| BX-SI055 | CCOC(=O)c2[nH]c1ccccc1c2N=NN3CCC(C)CC3 | 314.39 | 4.45 | 70.06 | 6 | 1 | 5 |
| BX-SI057 | CCOC(=O)c2[nH]c1ncccc1c2N=NN3CCN(C)CC3 | 316.37 | 2.29 | 86.19 | 8 | 1 | 5 |
| BX-SI058 | CCOC(=O)c2[nH]c1ncccc1c2N=NN3CCC(C)CC3 | 315.38 | 3.55 | 82.92 | 7 | 1 | 5 |

LogP – partition coefficient octanol/water, TPSA - topological polar surface area, nON – number of hydrogen-bond acceptors. nOHNH – number of hydrogen-bond donors, nRotB - number of rotatable bonds.

**Supplementary Table S2.** Yield and solubility of synthesized compounds in water.

| Compound | Yield, % | Melting point, ^0^C | Molar concentration,  mol/L | Mass concentration,  g/L | A (area of sample peak) | О (area of standard peak) |
| --- | --- | --- | --- | --- | --- | --- |
| BX-SI001 | 29 | 138 | 0.00028 | 0.090501 | 389241 | 4300962 |
| BX-SI003 | 37 | 147 | 0.00009 | 0.029646 | 109795 | 3703532 |
| BX-SI005 | 33 | 110 | 0.00021 | 0.070208 | 313745 | 4468779 |
| BX-SI010 | 19 | 118 | 0.00149 | 0.475472 | 1517469 | 3191497 |
| BX-SI016 | 28 | 140 | 0.01317 | 0.00001 | 18918 | 4004710 |
| BX-SI019 | 41 |  | 0.00029 | 0.088226 | 67738 | 767780 |
| BX-SI020 | 39 | 69 | 0.00002 | 0.007528 | 14418 | 1915143 |
| BX-SI021 | 44 | 78 | 0.00001 | 0.012458 | 28552 | 2291776 |
| BX-SI027 | 41 |  | 0.00278 | 0.927386 | 1582264 | 1706154 |
| BX-SI035 | 23 | 186 | 0.00035 | 0.011717 | 65906 | 624633 |
| BX-SI036 | 29 | 137 | 0.00003 | 0.010043 | 41175 | 4099932 |
| BX-SI037 | 37 | 162 | 0.00005 | 0.018354 | 65844 | 3587466 |
| BX-SI038 | 44 | 171 | 0.000001 | 0.000383 | 1416 | 3698917 |
| BX-SI039 | 39 | 176 | 0.00009 | 0.033074 | 104540 | 3160783 |
| BX-SI040 | 39 | 182 | 0.000014 | 0.00448 | 27015 | 6029484 |
| BX-SI043 | 39 | 193 | 0.000003 | 0.00088 | 4677 | 5314041 |
| BX-SI044 | 27 | 172 | 0.000005 | 0.01755 | 61670 | 3513898 |
| BX-SI045 | 36 | 183 | 0.00001 | 0.002772 | 8885 | 3205025 |
| BX-SI048 | 36 | 76 | 0.00009 | 0.034892 | 136328 | 3907157 |
| BX-SI055 | 28 | 177 | 0.000004 | 0.001216 | 5371 | 4416755 |
| BX-SI057 | 33 | 180 | 0.000053 | 0.016715 | 23959 | 1433390 |
| BX-SI058 | 35 | 240 | 0.000001 | 0.000399 | 541 | 1354526 |

**Supplementary Table S3.** Drug sensibility profile of MRSA clinical isolates.

| MRSA strain | CEF-CIP-CLIND-ERI-GEN-SUL-TET | Daptomycine | Fusidic acid | Linezolid | Mupirocin | Tedizolid | Telavancin | Tigecycline | Vancomycin |
| --- | --- | --- | --- | --- | --- | --- | --- | --- | --- |
| 1 | S S S S S R R | S | S | S | S | N/D | N/D | S | S |
| 2 | S R R R R S R | S | S | S | S | N/D | N/D | R | S |
| 3 | S R S S R S S | S | S | S | S | N/D | N/D | S | S |
| 4 | S R S S R S S | S | S | S | S | N/D | N/D | S | S |
| 5 | S R R R R S S | S | S | S | S | N/D | N/D | S | S |
| 6 | R R R R S S S | S | S | S | S | S | N/D | S | S |
| 7 | S R R R R S R | S | S | S | S | S | N/D | S | S |
| 8 | R R S R R S R | S | S | S | S | S | N/D | R | S |
| 9 | S R S R R S R | S | S | S | S | S | N/D | R | S |
| 10 | S R S S S S S | S | S | S | S | S | N/D | S | S |
| 12 | S R R R S S R | S | S | S | S | S | N/D | R | S |
| 13 | R R S S R S R | S | S | S | S | S | N/D | R | S |
| 14 | S R R R R S S | S | S | S | S | S | N/D | S | S |
| 15 | S S S S S S R | S | S | S | S | S | N/D | S | S |
| 16 | S R R R R S S | S | S | S | S | S | N/D | S | S |
| 17 | S R S S R I S | S | R | S | S | S | N/D | R | S |
| 18 | S R S S R S S | S | S | S | S | S | N/D | S | S |
| 21 | R R R R S S R | S | S | S | S | S | N/D | S | S |
| 23 | S S R R R S S | S | S | S | S | S | N/D | S | S |
| 24 | S R S S R S S | S | S | S | S | S | N/D | S | S |
| 25 | S S S S R S S | N/D | S | S | S | S | S | S | S |
| 26 | S S S S S S S | N/D | S | S | S | S | S | S | S |
| 27 | R R S S R S R | N/D | S | S | S | S | S | S | S |
| 28 | S R S R R S R | N/D | S | S | S | S | S | S | S |
| 29 | S R S S S S S | N/D | S | S | S | S | S | S | S |
| 30 | S R S I S S S | S | S | S | S | N/D | N/D | S | S |
| 31 | R R R R R S S | S | S | S | S | N/D | N/D | S | S |
| 34 | R R S I R S S | S | S | S | S | N/D | N/D | S | S |
| 35 | R R S I R S S | S | S | S | S | N/D | N/D | S | S |
| 36 | S S S S S S S | N/D | S | S | S | S | S | S | S |
| 40 | S R R R R S R | S | S | S | S | N/D | N/D | S | S |
| 41 | R R S S R S R | N/D | S | S | S | S | S | S | S |
| 42 | S R S S R S S | N/D | S | S | S | S | S | S | S |
| 43 | S R S I R S S | S | S | S | S | N/D | N/D | S | S |
| 44 | R R S I R S S | N/D | S | S | S | S | S | S | S |
| 45 | S R R R R S I | N/D | S | S | S | S | S | S | S |
| 46 | S R S S S S S | S | S | S | S | N/D | N/D | S | S |
| 48 | R R R R R S R | S | S | S | S | N/D | N/D | S | S |
| 49 | S S S I S S R | S | S | S | S | N/D | N/D | S | S |
| 50 | R S R R R S R | S | S | S | I | N/D | N/D | S | S |
| 51 | S R S S R S S | N/D | S | S | S | S | S | S | S |

CEF – cefotaxime, CIP – ciprofloxacin, CLIND – clindamycin, ERI – erythromycin, GEN – gentamicin, SUL – sulfamethoxazole, TET – tetracycline; S – sensitive, R – resistant, I – intermediate according to EUCAST guidelines, N/D – no data.

**Supplementary Table S4.** MIC of BX-SI043 vs BX-SI001 on 51 clinical isolate.

| MRSA strain number | MIC (mg/L) | | MRSA strain number | MIC (mg/L) | |
| --- | --- | --- | --- | --- | --- |
|  | BX-SI001 | BX SI043 |  | BX-SI001 | BX SI043 |
| 1 | 0.5 | 0.25 | 26 | 0.5 | 0.25 |
| 2 | 1 | 0.5 | 27 | 1 | 0.5 |
| 3 | 1 | 0.5 | 28 | 1 | 0.5 |
| 4 | 1 | 0.5 | 29 | 1 | 0.5 |
| 5 | 0.5 | 0.25 | 30 | 1 | 0.5 |
| 6 | 0.5 | 0.5 | 31 | 1 | 0.5 |
| 7 | 1 | 0.5 | 34 | 1 | 0.125 |
| 8 | 1 | 0.25 | 35 | 1 | 0.5 |
| 9 | 1 | 0.5 | 36 | 1 | 0.25 |
| 10 | 1 | 0.25 | 40 | 1 | 0.5 |
| 12 | 1 | 0.25 | 41 | 1 | 0,25 |
| 13 | 1 | 0.5 | 42 | 1 | 0.5 |
| 14 | 1 | 0.25 | 43 | 1 | 0.5 |
| 15 | 1 | 0.25 | 44 | 1 | 0.5 |
| 16 | 0.5 | 0.25 | 45 | 1 | 0.5 |
| 17 | 1 | 0.5 | 46 | 1 | 0.5 |
| 18 | 1 | 0.5 | 48 | 1 | 0.5 |
| 21 | 1 | 0.5 | 49 | 1 | 0.25 |
| 23 | 1 | 0.5 | 50 | 1 | 0.5 |
| 24 | 1 | 0.5 | 51 | 1 | 0.5 |
| 25 | 1 | 0.5 |  |  |  |

**Supplementary Table S5.** Stability of ВХ-SI043 and verapamil used as a control in the microsomal human and rat liver microsomes.

|  | Human liver microsomes | | | Rat liver microsomes | | |
| --- | --- | --- | --- | --- | --- | --- |
|  | t_1/2,_ min | Cl_int,_ μl/min/mg protein | Remaining after  30 min, % | t_1/2,_ min | Cl_int,_ μl/min/mg protein | Remaining after  30 min, % |
| ВХSI043 | 2.73 | 1015 | 0.00 | 1.30 | 2138 | 0.00 |
| Verapamil | 29.9 | 92.8 | 50.7 | 14.0 | 199 | 23.2 |

**Supplementary Table S6.** Inhibition of the cytochrome activity in the microsomal liver fraction by BX-SI043 and corresponding isoform inhibitors.

| Cytochrome p450 isoforms | 1A2 | 2C19 | 3A4-M | 3A4-T | 2C9 | 2C8 | 2D6 |
| --- | --- | --- | --- | --- | --- | --- | --- |
| Inhibitors | α-naphthoflavone | Fluvoxamine | Ketoconazole | | Sulfafenazole | Quercetin | Quinidine |
| IC_50_, µM | 0.011 | 0.37 | 0.07 | 0.04 | 0.546 | 2.604 | 0.285 |
| BX-SI043,  IC_50_, µM | N/A | N/A | N/A | N/A | N/A | N/A | N/A |

**Supplementary Table S7.** Permeability and asymmetry of transport of test and control compounds in the Caco-2 cell model.

| Compound | P_app_ A-B, 10^-6^ sm/s | SD  (A-B) | P_app_ B-A, 10^-6^ sm/s | SD  (B-A) | Asymmetry index | Mass balance |
| --- | --- | --- | --- | --- | --- | --- |
| ВХ-SI043 | 0.51 | 0.03 | 0.19 | 0.01 | 0.37 | 49.0/25.1 |
| Ranitidine | 1.48 | 0.13 | –* | – | – | 96.0 |
| Propranolol | 44.6 | 1.94 | –* | – | – | 91.7 |
| Rhodamine | 1.14 | 0.12 | 3.65 | 0.42 | 3.20 | 92.1/95.8 |
| Rhodamine+ cyclosporin A | 0.89 | 0.07 | 0.97 | 0.12 | 1.09 | 91.5/84.6 |

* - for these compounds the asymmetry of transport is known to be absent and therefore the asymmetry was not evaluated.

**Supplementary Table S8.** Effect of the test substance on body weights of experimental animals at intragastric administration.

| Male | Groups | Dose | Group number |  | Experiment day | | | |
| --- | --- | --- | --- | --- | --- | --- | --- | --- |
|  |  |  |  |  | 1 | 2 | 7 | 15 |
|  | Starch solution, 1% | 0 mg/kg | 1 | M±SEM, g | 195,3±2,87 | 209,5±6,76 | 256,8±5,23 | 281,0±5,29 |
|  |  |  |  | n | 4 | 4 | 4 | 4 |
|  | Tested substance | 300 mg/kg | 2 | M±SEM, g | 202,8±2,87 | 205,7±1,73 | 256,7±4,65 | 280,8±8,21 |
|  |  |  |  | n | 6 | 6 | 6 | 6 |
|  |  | 600 mg/kg | 5 | M±SEM, g | 201,3±2,80 | 222,2±2,63 | 253,3±4,15 | 274,3±6,51 |
|  |  |  |  | n | 6 | 6 | 6 | 6 |
|  |  | 1000 mg/kg | 4 | M±SEM, g | 191,0±5,79 | 206,5±6,61 | 231,8±12,48 | 273,0±11,56 |
|  |  |  |  | n | 6 | 6 | 5 | 5 |
|  |  | 2000 mg/kg | 3 | M±SEM, g | 196,0±1,61 | 212,7±3,91 | 236,0±1,53 | 259,7±3,18 |
|  |  |  |  | n | 6 | 6 | 3 | 3 |
| Female | Starch solution, 1% | 0 mg/kg | 1 | M±SEM, g | 186,0±4,60 | 196,3±6,05 | 215±6,72 | 229,8±6,8 |
|  |  |  |  | n | 4 | 4 | 4 | 4 |
|  | Tested substance | 300 mg/kg | 2 | M±SEM, g | 195,3±3,67 | 194,8±3,92 | 220,0±7,86 | 234,7±9,33 |
|  |  |  |  | n | 6 | 6 | 6 | 6 |
|  |  | 600 mg/kg | 5 | M±SEM, g | 195,2±4,56 | 207,7±4,08 | 222,5±5,43 | 221,2±5,98 |
|  |  |  |  | n | 6 | 6 | 6 | 6 |
|  |  | 1000 mg/kg | 4 | M±SEM, g | 185,7±4,61 | 192,2±3,94 | 189,6±6,16 | 199,2±6,46 |
|  |  |  |  | n | 6 | 6 | 5 | 5 |
|  |  | 2000 mg/kg | 3 | M±SEM, g | 196,5±2,06 | 205,8±1,64 | 183,5±11,99 | 210,8±5,91 |
|  |  |  |  | n | 6 | 6 | 6 | 5 |

**Supplementary Table S9.** Effect of the test substance on relative organ weight of experimental animals after 15 days of the study, % of body weight, (M±SEM).

| Sex | Parameters | Starch Solution 1%, mg/kg | Tested substance, mg/kg | | | |
| --- | --- | --- | --- | --- | --- | --- |
|  |  | 0 | 300 | 600 | 1000 | 2000 |
|  |  | Group №1 | Group №2 | Group №5 | Group №4 | Group №3 |
|  | n | 4 | 6 | 6 | 5 | 3 |
| Male | Heart | 0,45±0,028 | 0,45±0,012 | 0,50±0,013 | 0,51±0,022 | 0,48±0,046 |
|  | Lungs with trachea | 0,72±0,034 | 0,71±0,034 | 0,86±0,108 | 1,00±0,096 | 0,75±0,037 |
|  | Thymus | 0,248±0,0438 | 0,246±0,0276 | 0,27±0,0191 | 0,278±0,0278 | 0,20±0,0116 |
|  | Liver | 4,20±0,375 | 4,53±0,26 | 4,69±0,216 | 4,91±0,128 | 4,08±0,252 |
|  | Spleen | 0,29±0,027 | 0,34±0,017 | 0,32±0,036 | 0,32±0,031 | 0,28±0,018 |
|  | Kidneys | 0,91±0,080 | 0,98±0,046 | 0,94±0,031 | 0,87±0,008 | 0,90±0,034 |
|  | Adrenal glands | 0,021±0,0024 | 0,022±0,0012 | 0,028±0,0017 | 0,027±0,0022 | 0,026±0,0014 |
|  | Brain | 0,72±0,025 | 0,77±0,020 | 0,77±0,013 | 0,77±0,036 | 0,78±0,024 |
|  | Testes | 1,22±0,020 | 1,17±0,039 | 1,28±0,033 | 1,11±0,064 | 1,2±0,120 |
| Female | Heart | 0,44±0,009 | 0,46±0,012 | 0,48±0,007 | 0,52±0,02 | 0,41±0,079 |
|  | Lungs with trachea | 0,79±0,025 | 0,77±0,037 | 0,82±0,044 | 0,79±0,057 | 0,9±0,07 |
|  | Thymus | 0,281±0,0255 | 0,280±0,016 | 0,310±0,013 | 0,270±0,020 | 0,310±0,032 |
|  | Liver | 4,12±0,215 | 4,79±0,090 | 4,64±0,067 | 4,67±0,23 | 4,73±0,167 |
|  | Spleen | 0,32±0,033 | 0,37±0,033 | 0,33±0,045 | 0,42±0,05 | 0,38±0,028 |
|  | Kidneys | 0,82±0,044 | 0,87±0,065 | 0,78±0,021 | 0,82±0,027 | 0,89±0,033 |
|  | Adrenal glands | 0,03±0,0014 | 0,03±0,003 | 0,04±0,002 | 0,04±0,004 | 0,04±0,004 |
|  | Brain | 0,92±0,052 | 0,84±0,032 | 0,86±0,031 | 0,97±0,022 | 0,93±0,027 |
|  | Ovaries | 0,070±0,010 | 0,067±0,0043 | 0,066±0,0032 | 0,085±0,0075 | 0,088±0,0092 |
