# Supplementary figures and images for: Novel Triazeneindole Antibiotics: Synthesis and Hit-to-Lead Optimization
Source: Int J Mol Sci. 2025 Feb 21;26(5):1870. doi: 10.3390/ijms26051870 (PMC11899342; doi:10.3390/ijms26051870)

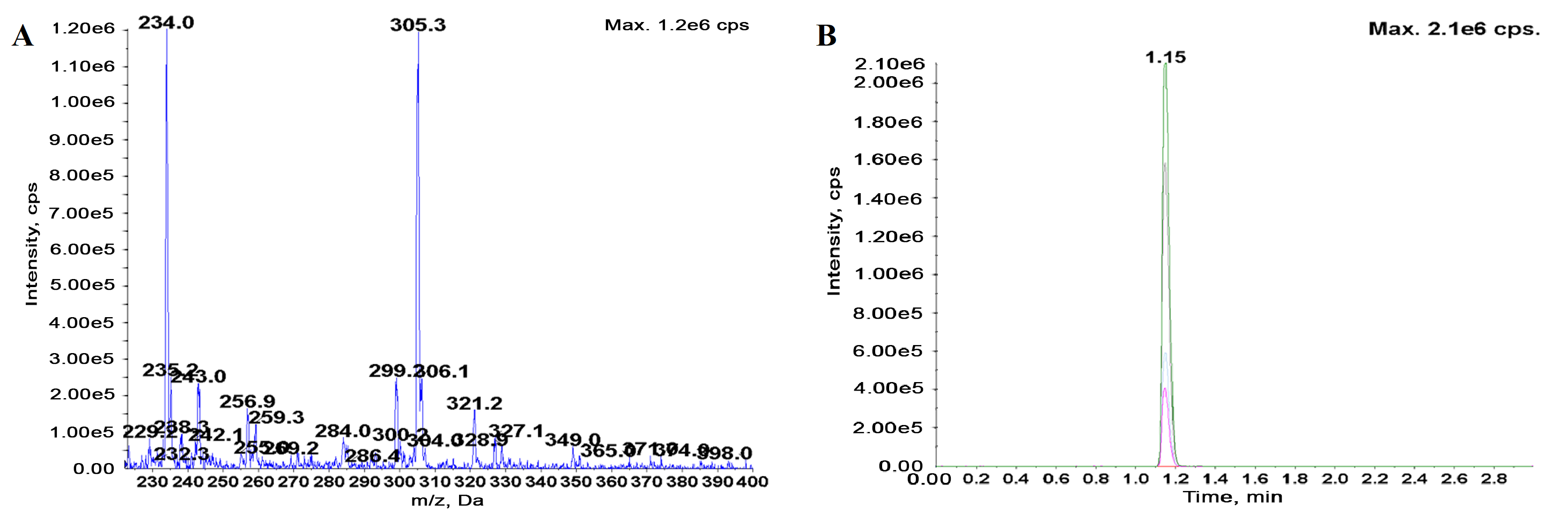

Supplement: Supplementary file 1 [file ijms-26-01870-s001.zip › Supplementary Figure S1.png]

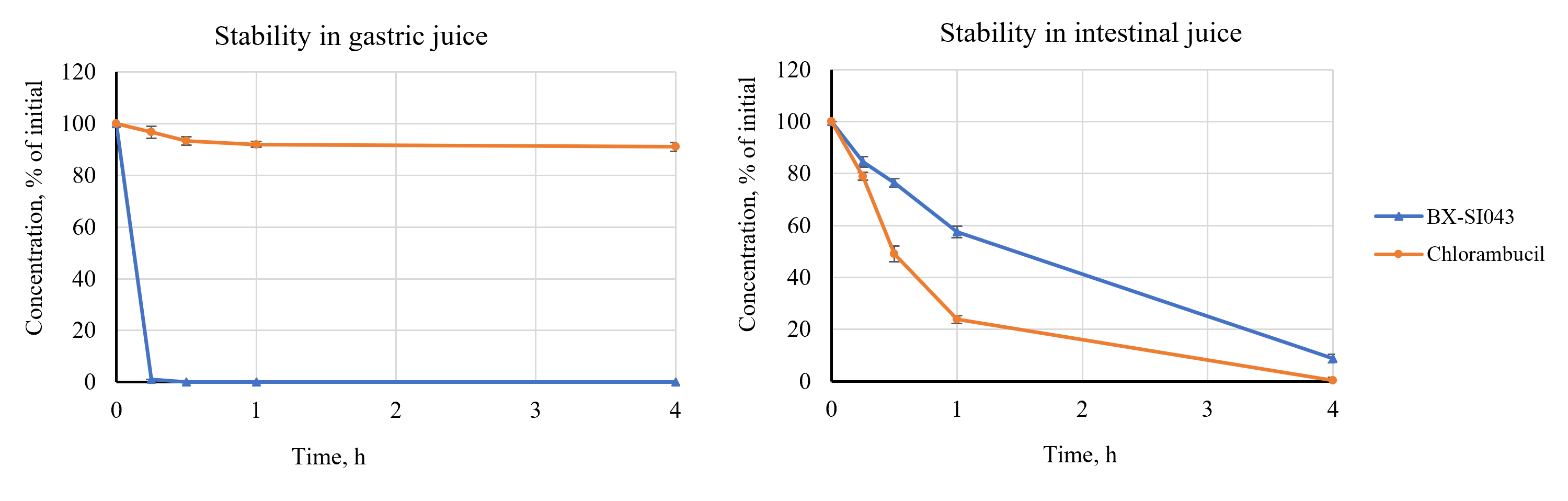

Supplement: Supplementary file 1 [file ijms-26-01870-s001.zip › Supplementary Figure S2.png]

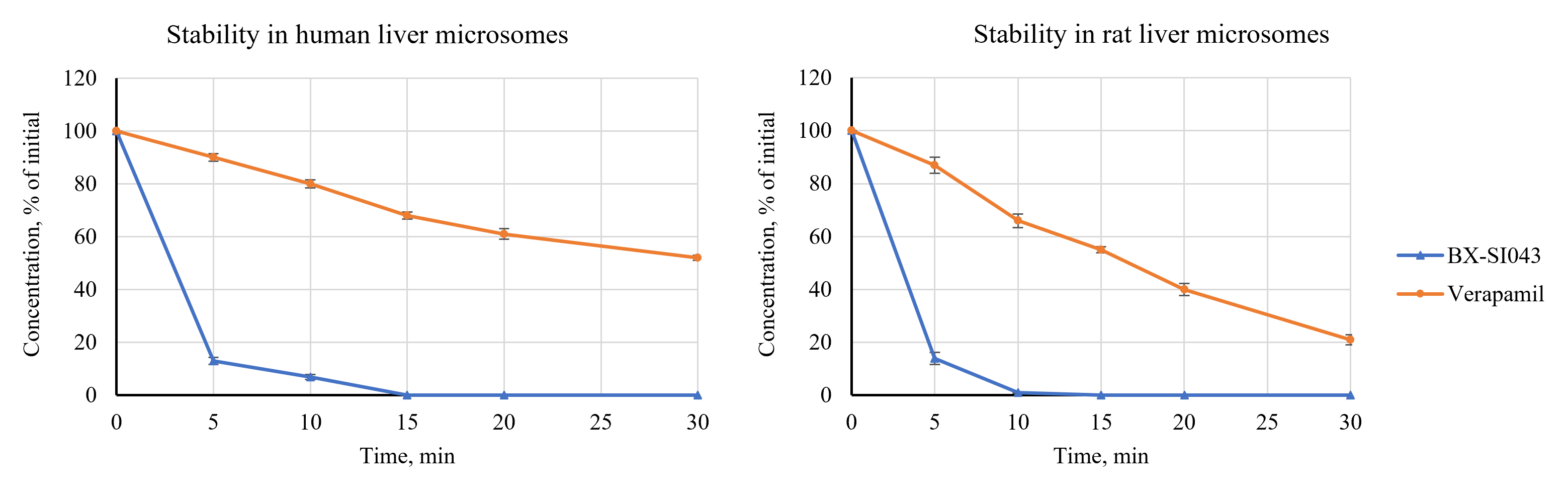

Supplement: Supplementary file 1 [file ijms-26-01870-s001.zip › Supplementary Figure S3.png]

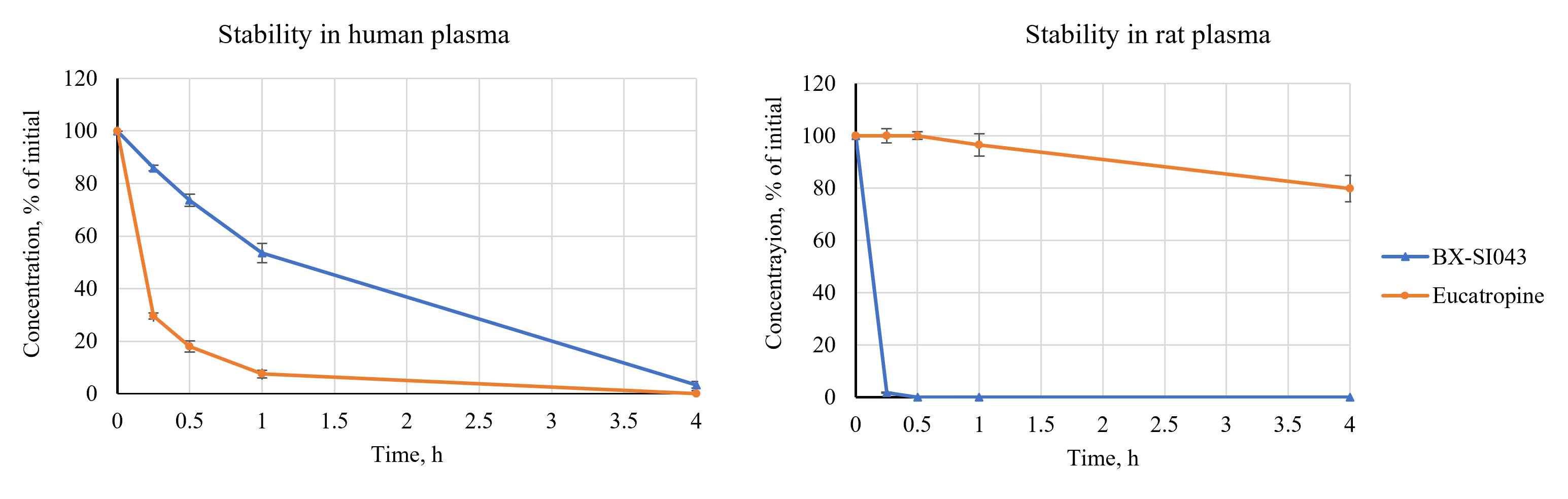

Supplement: Supplementary file 1 [file ijms-26-01870-s001.zip › Supplementary Figure S4.png]

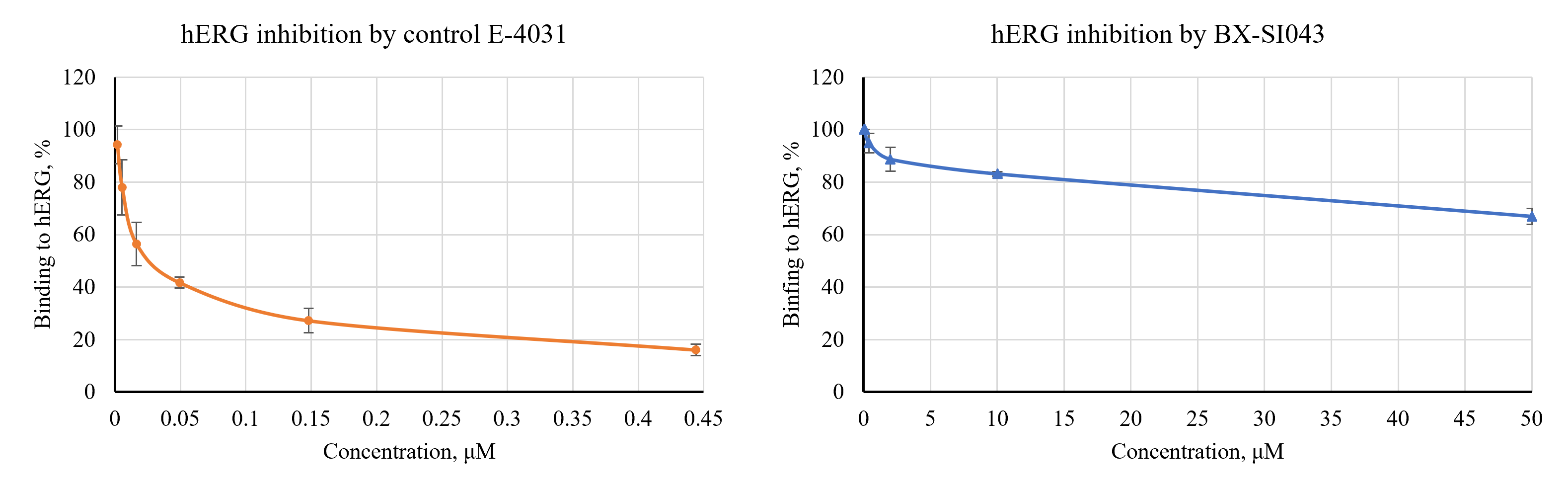

Supplement: Supplementary file 1 [file ijms-26-01870-s001.zip › Supplementary Figure S5.png]

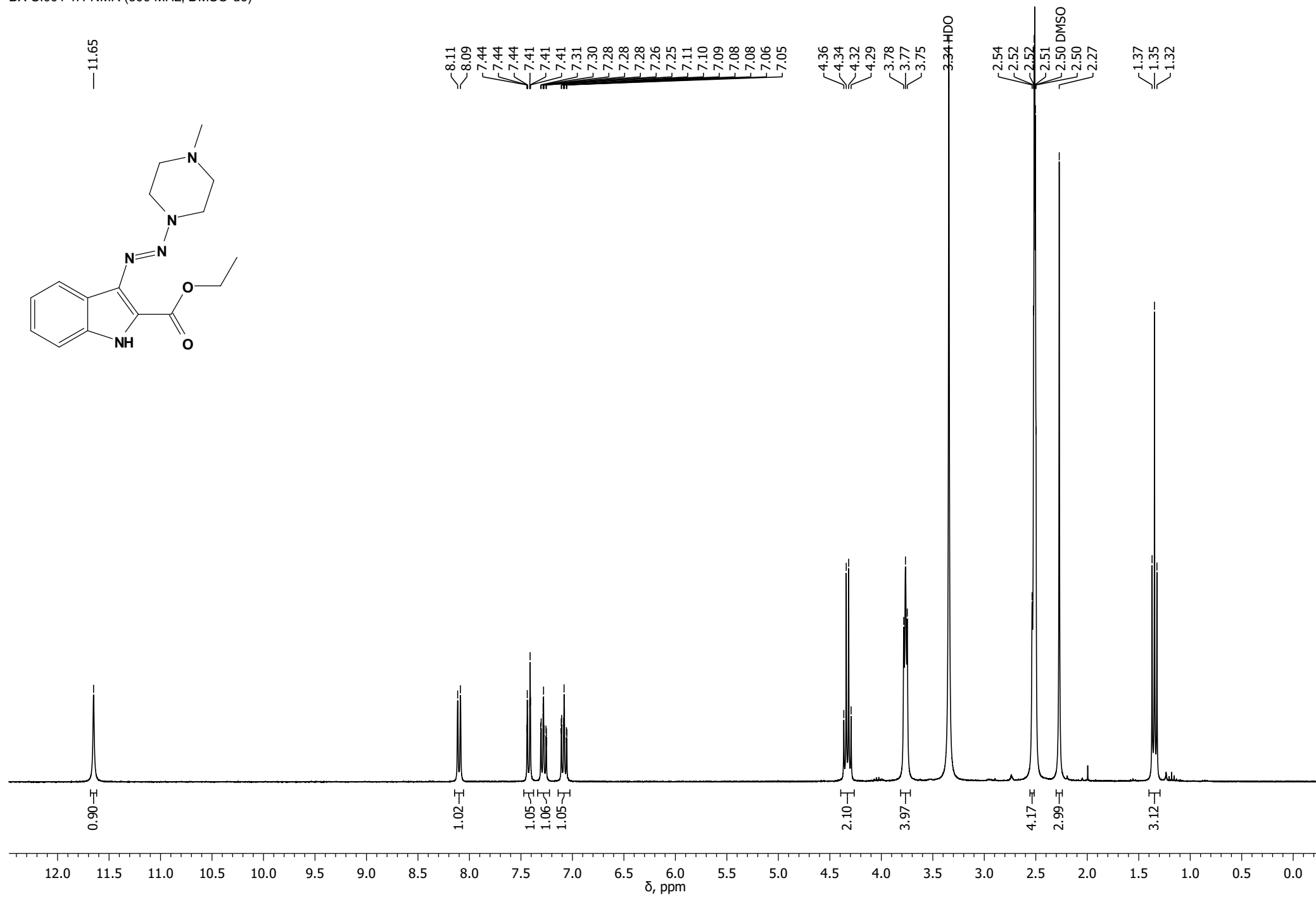

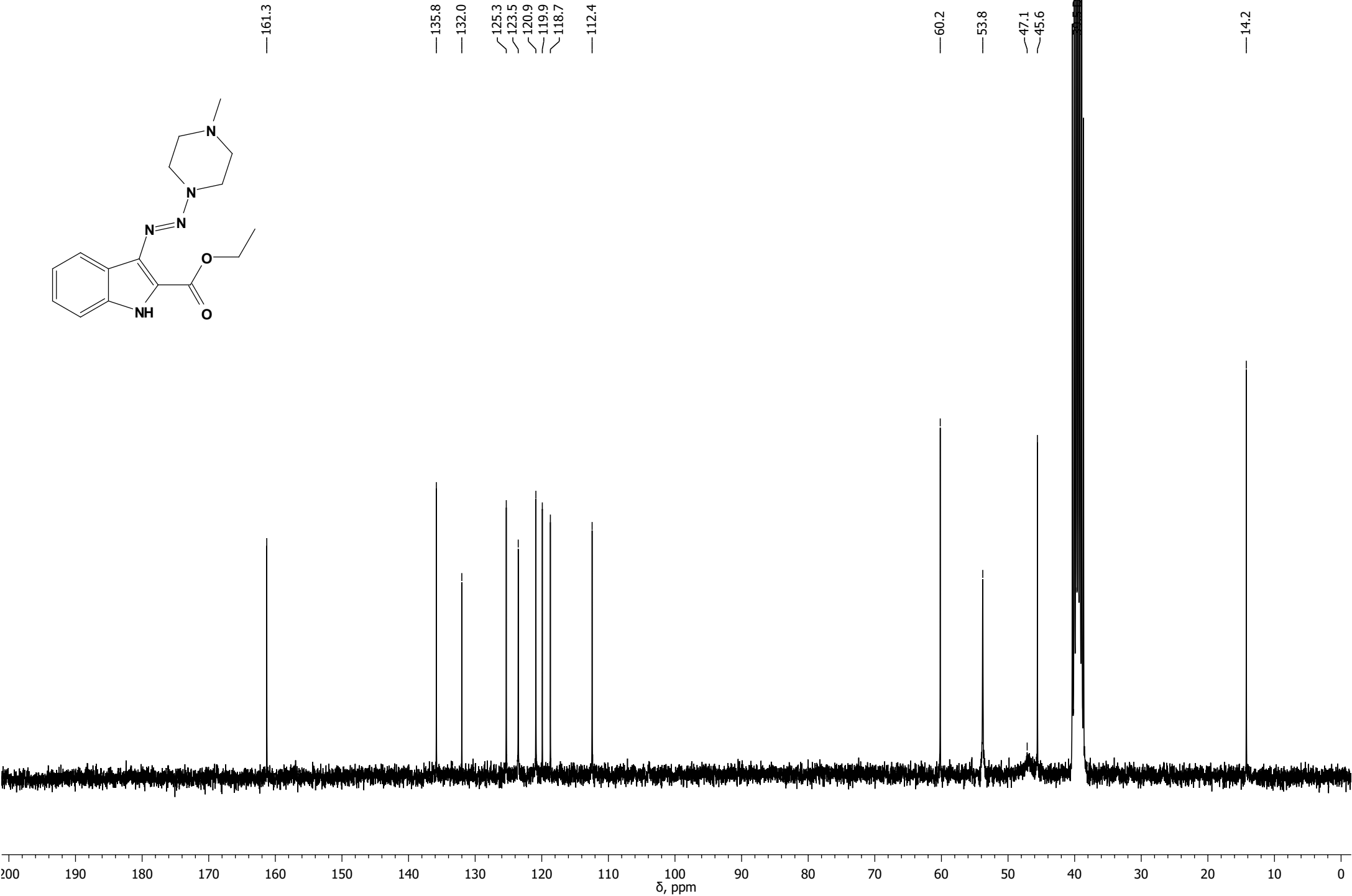

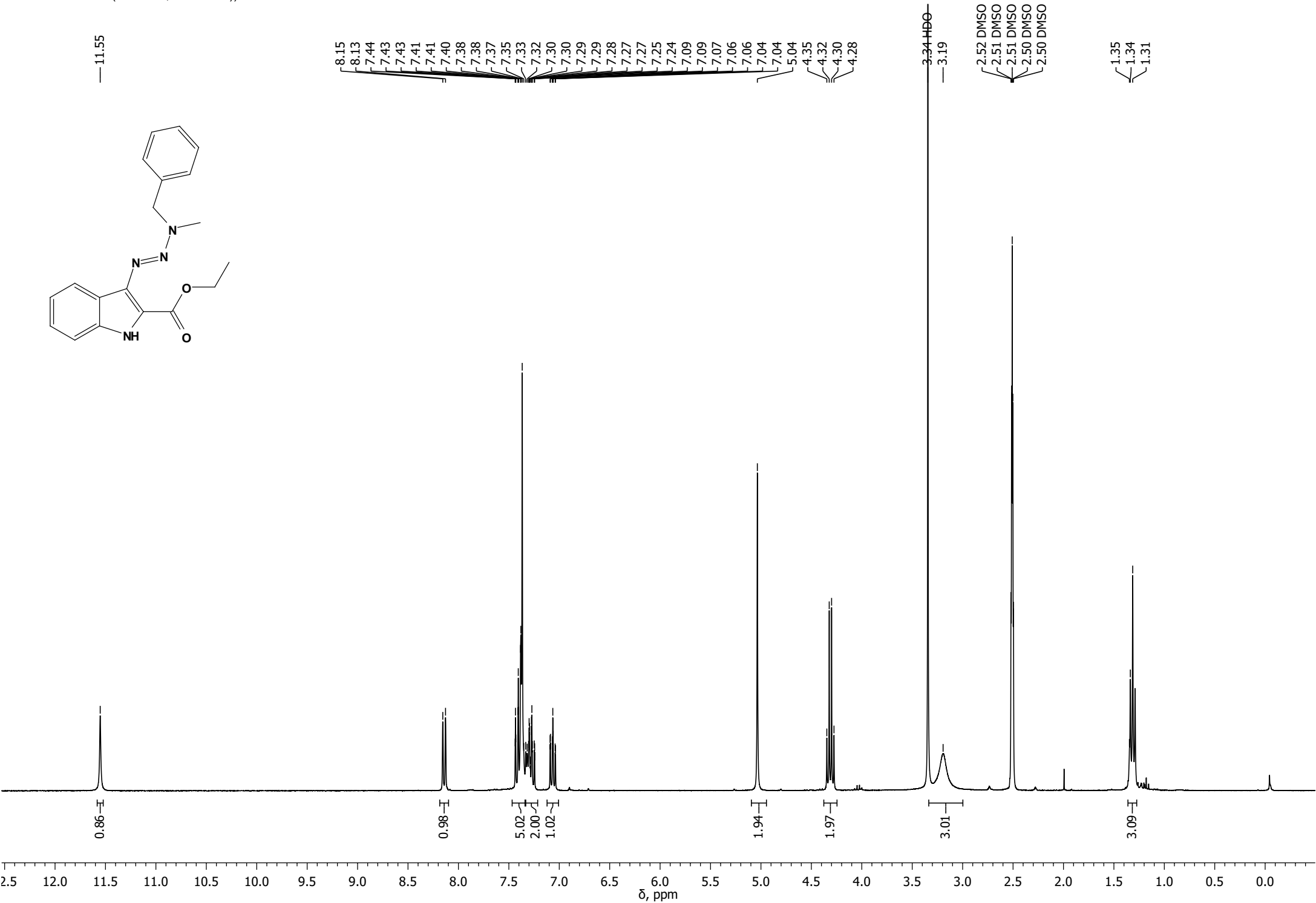

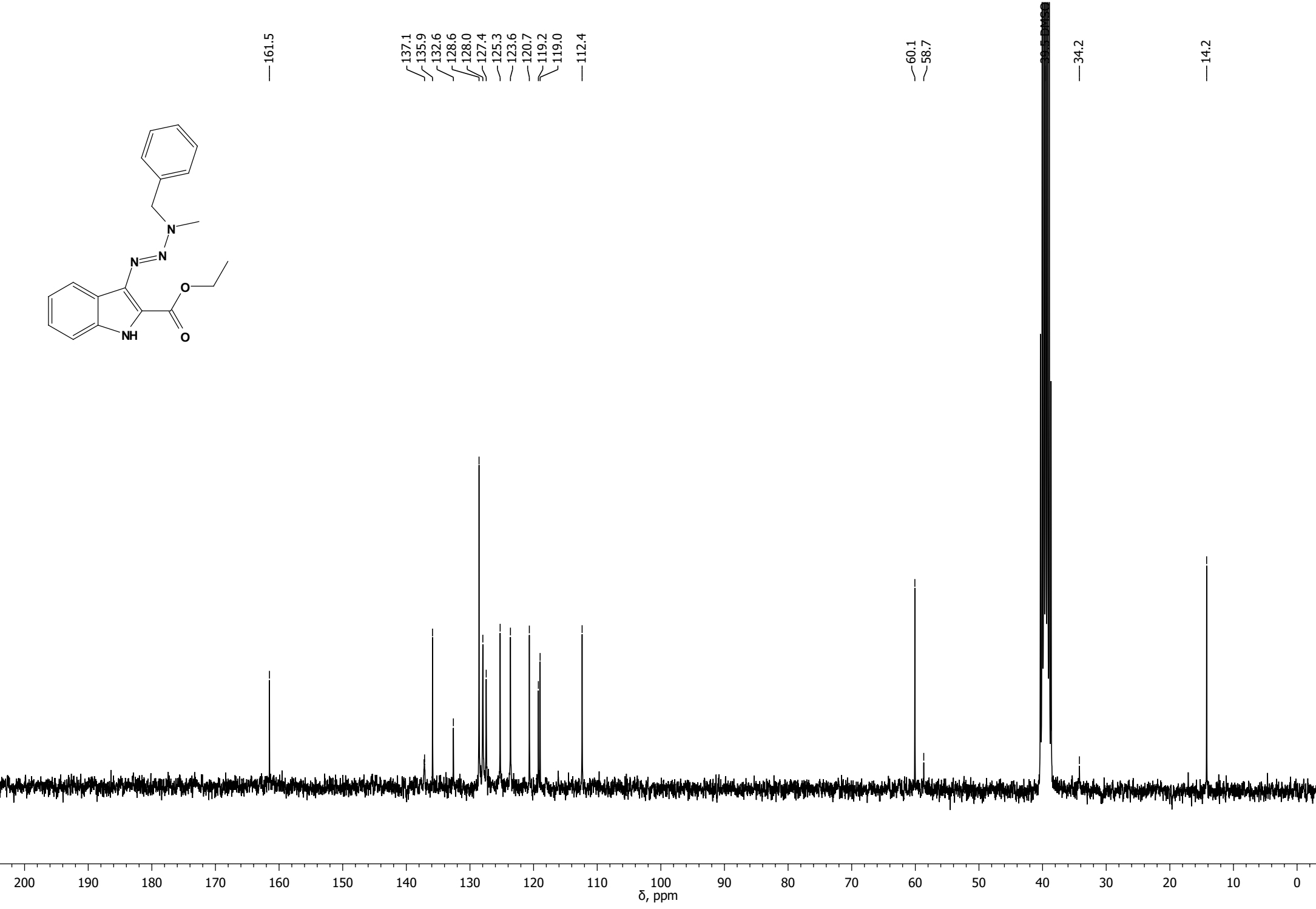

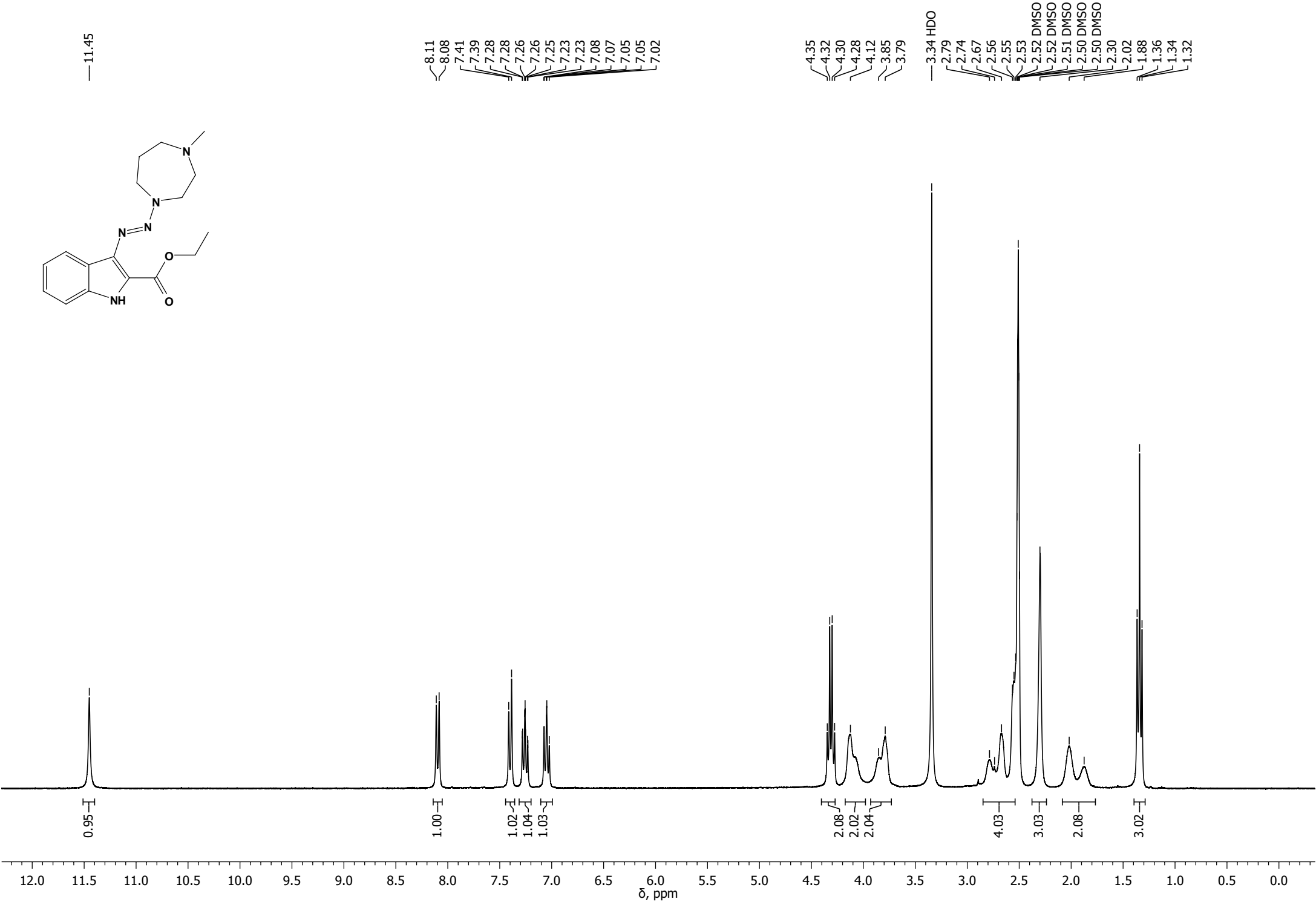

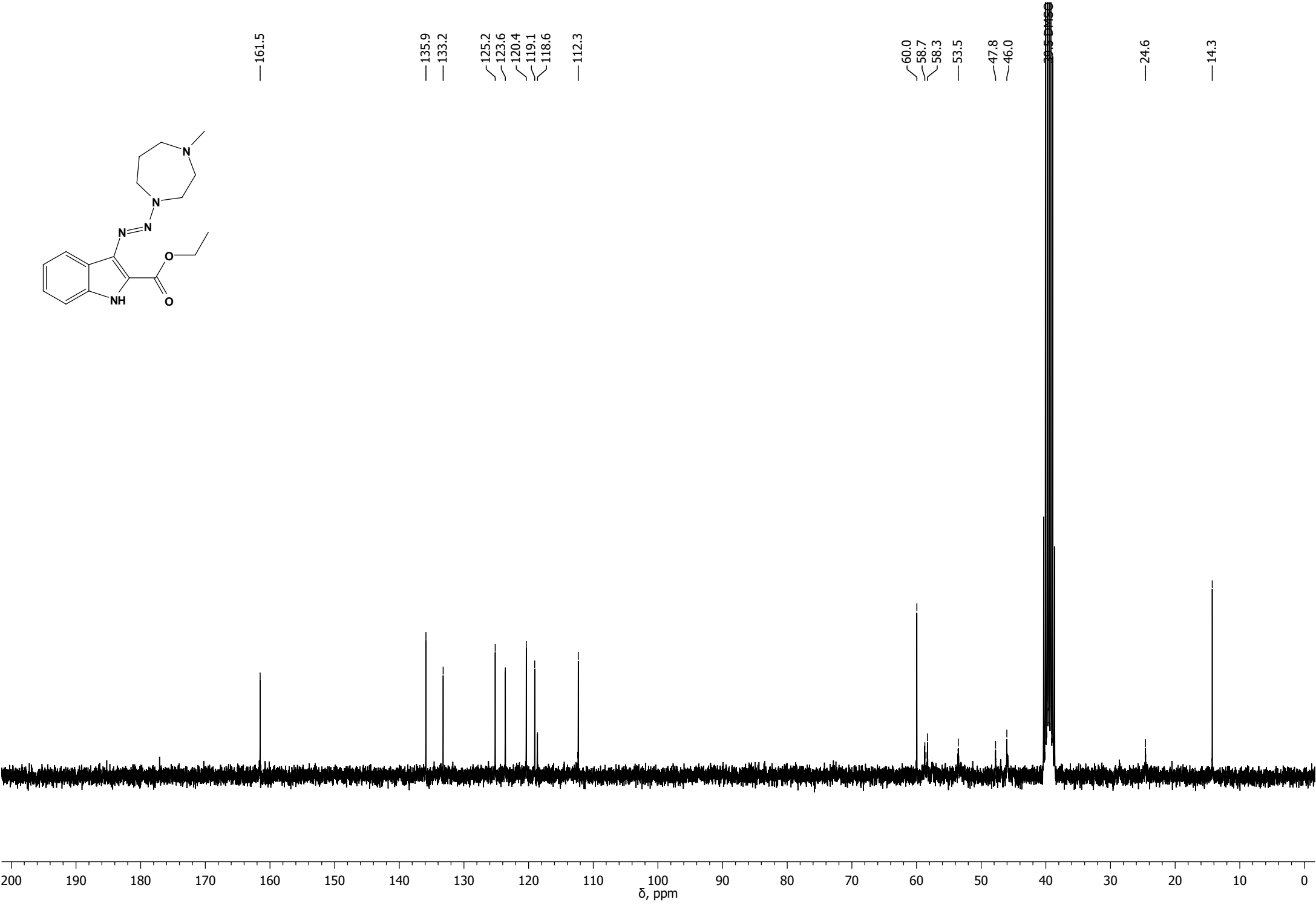

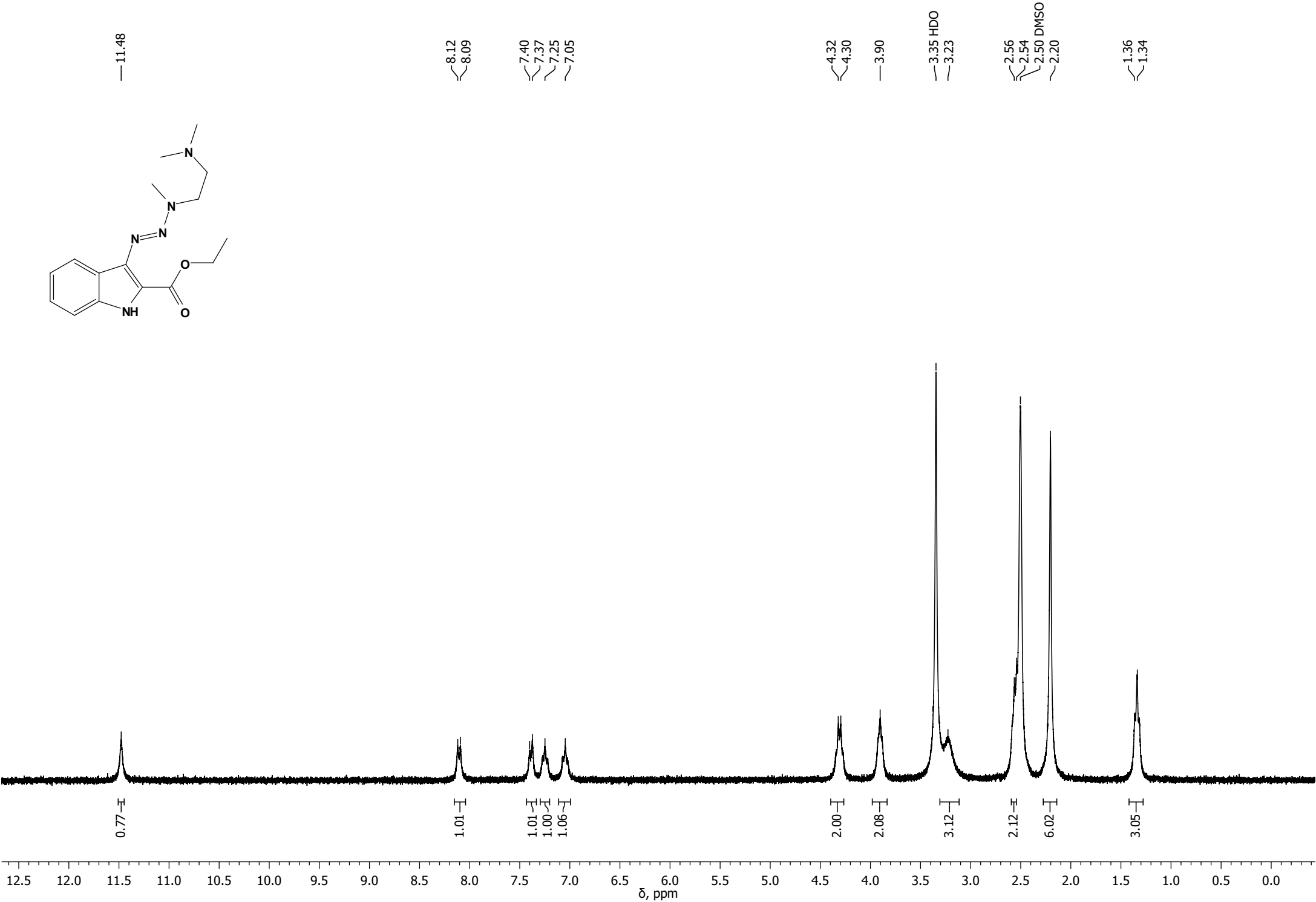

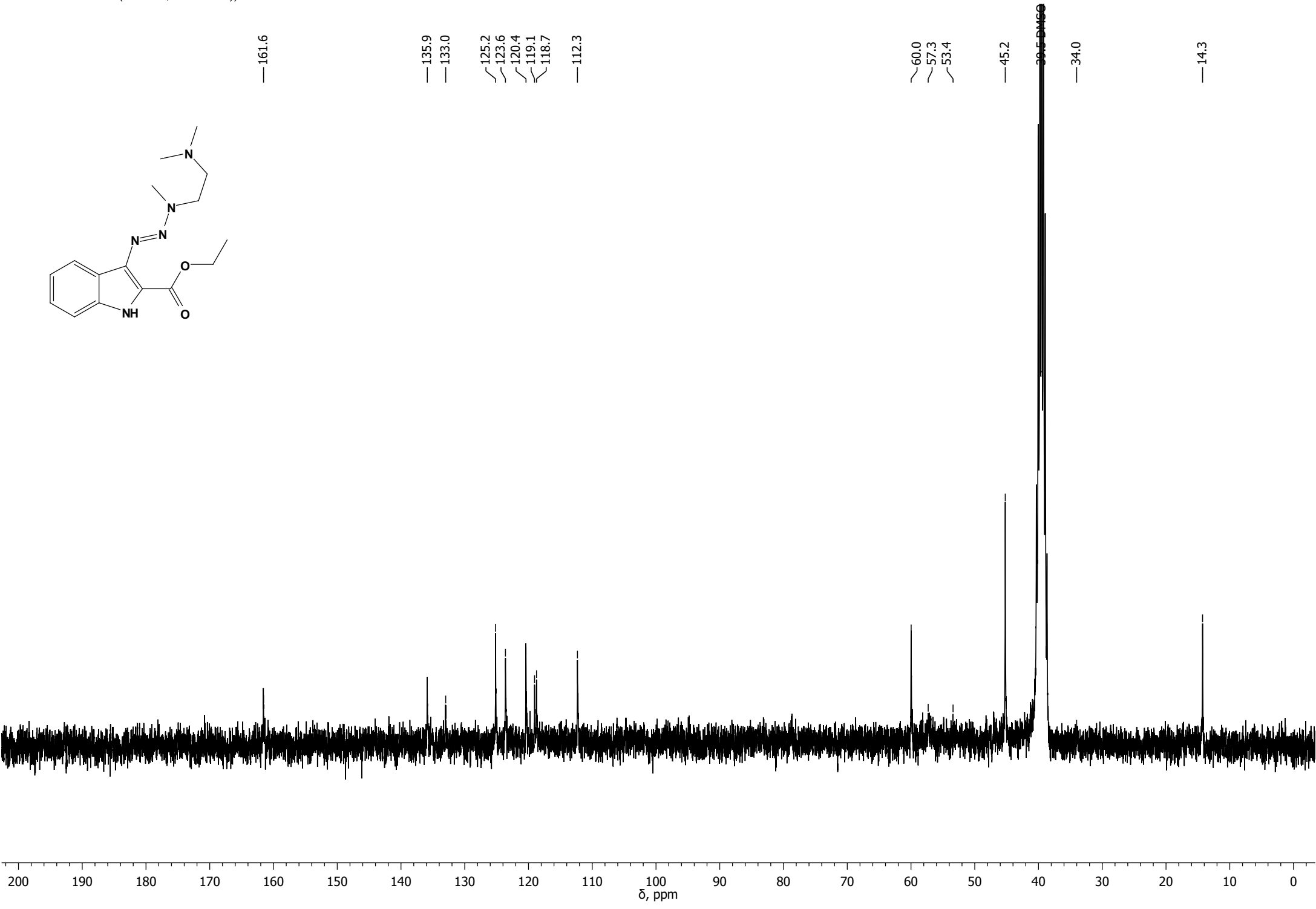

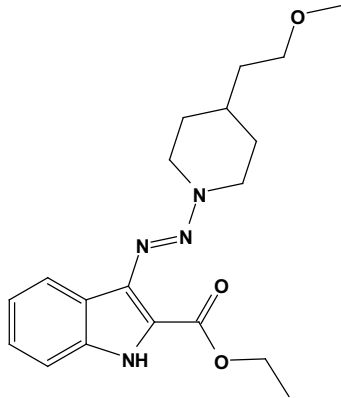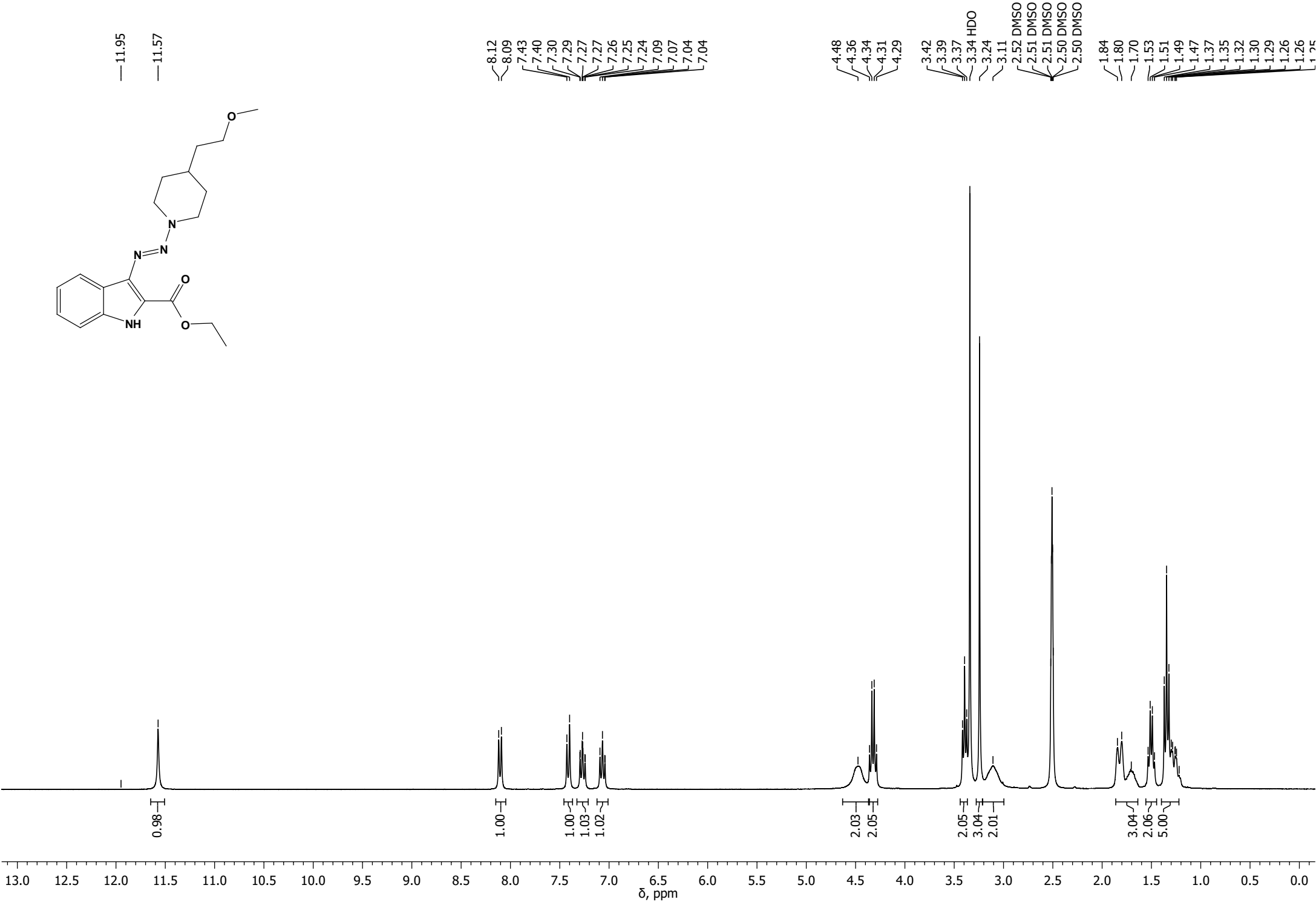

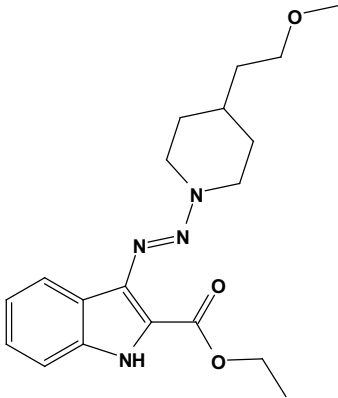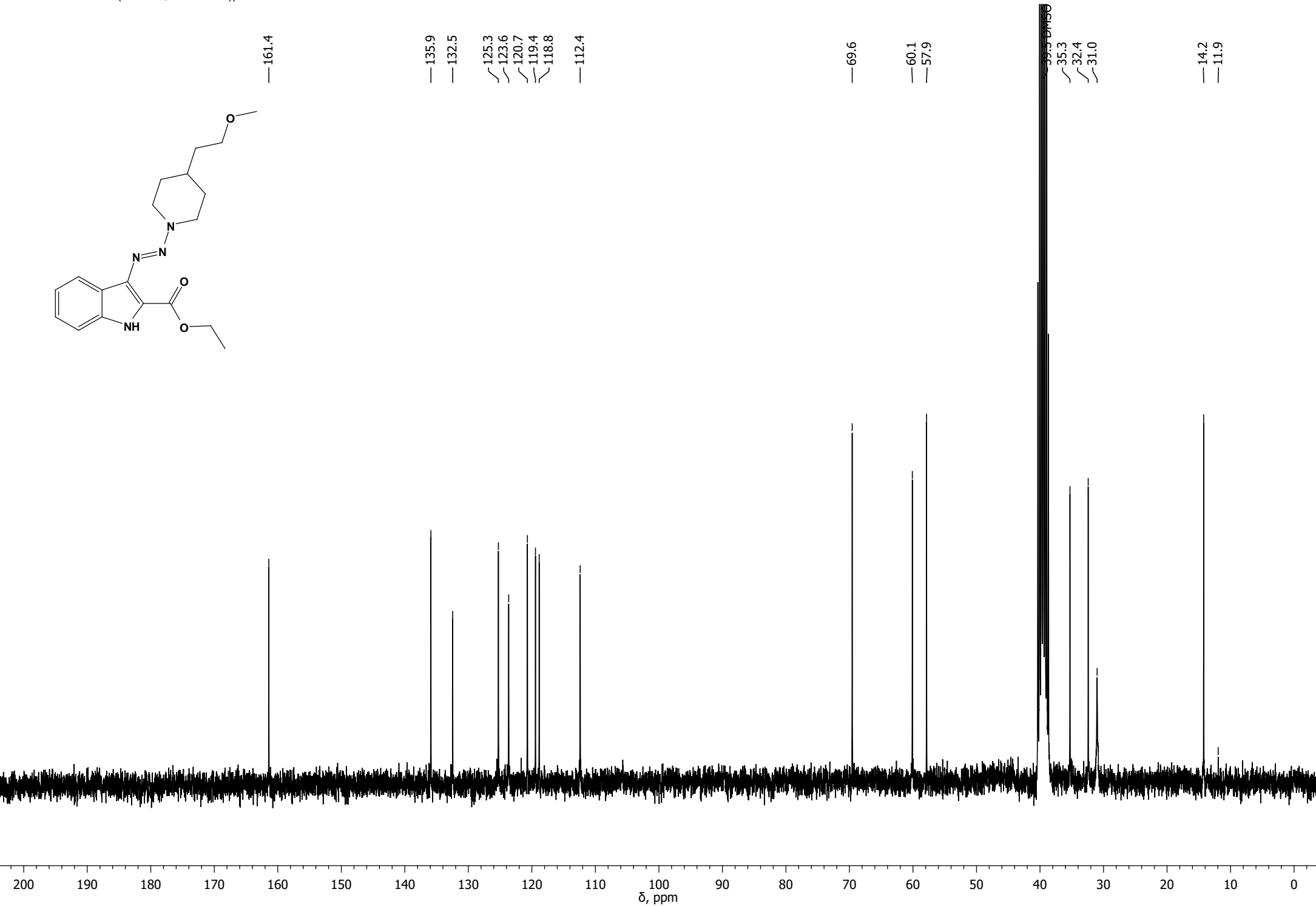

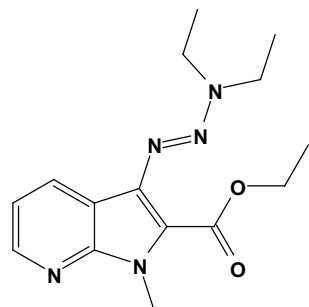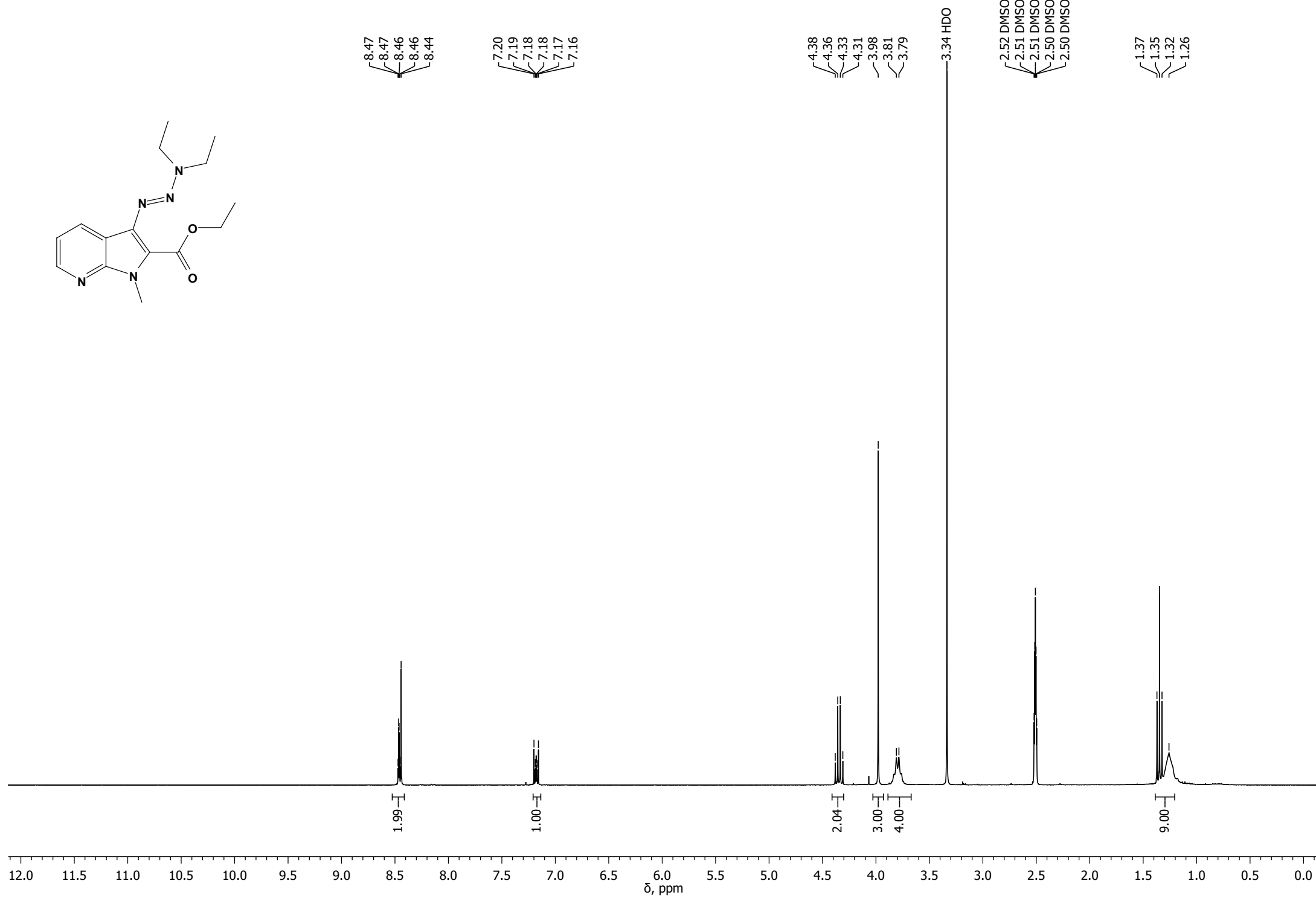

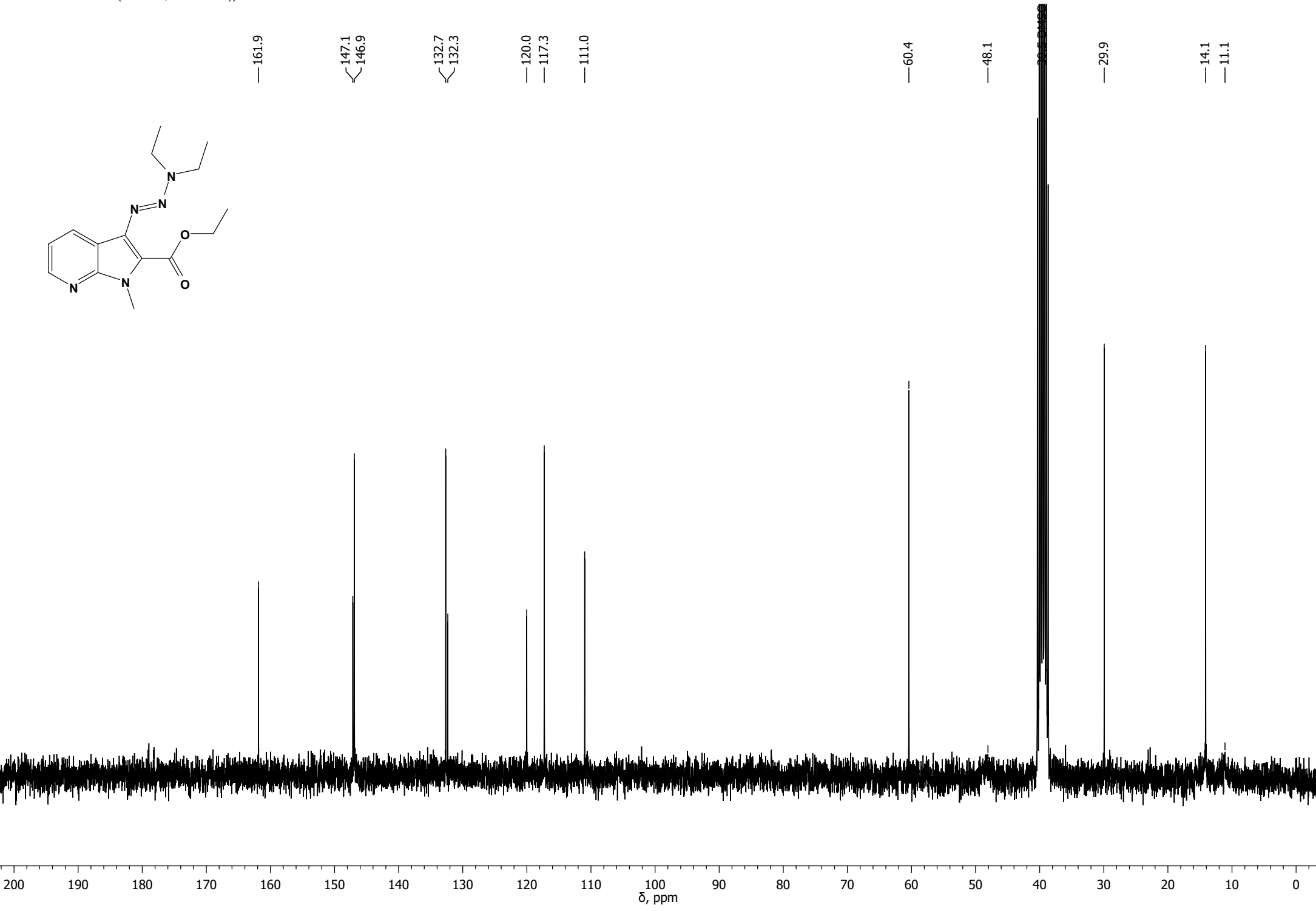

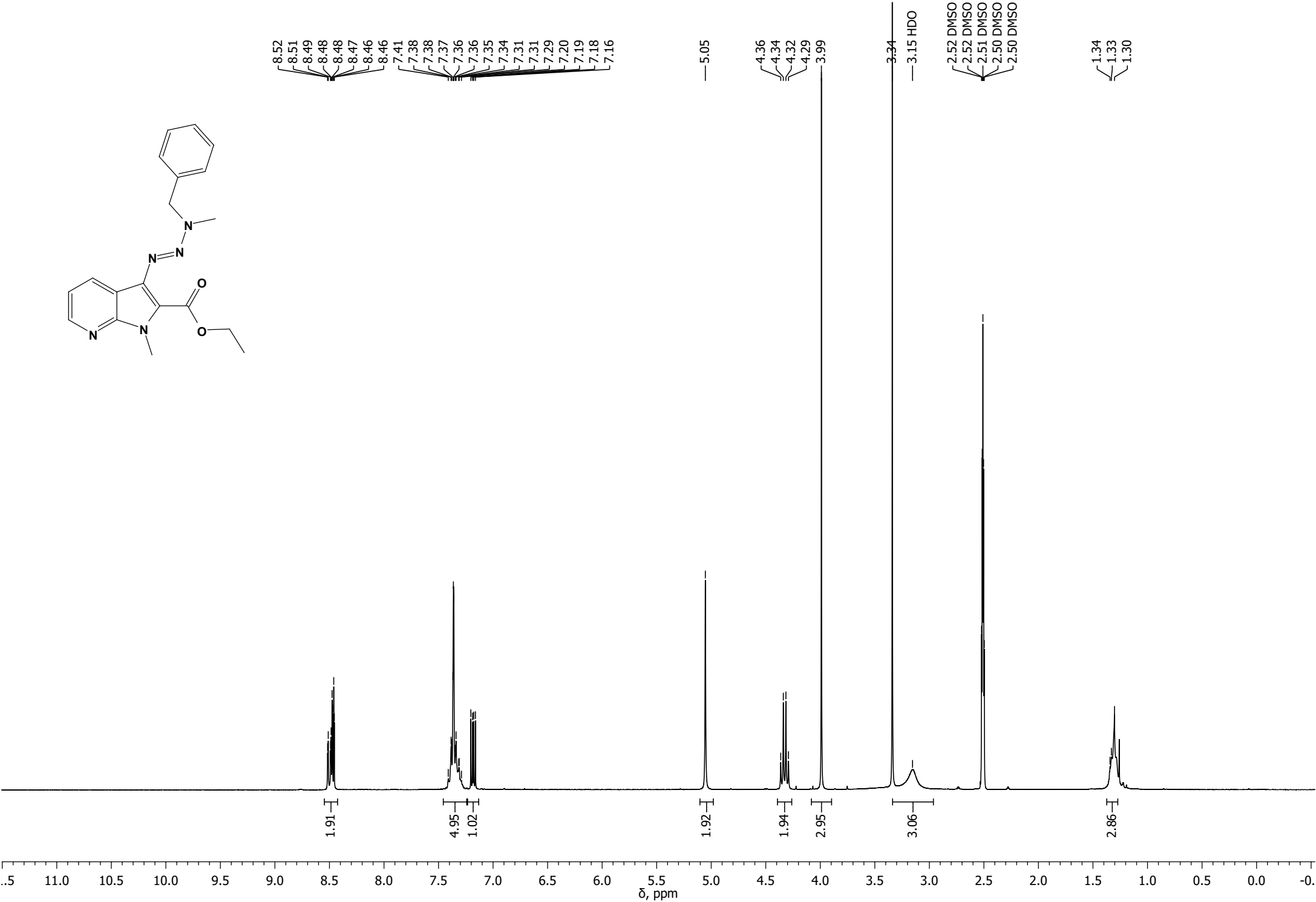

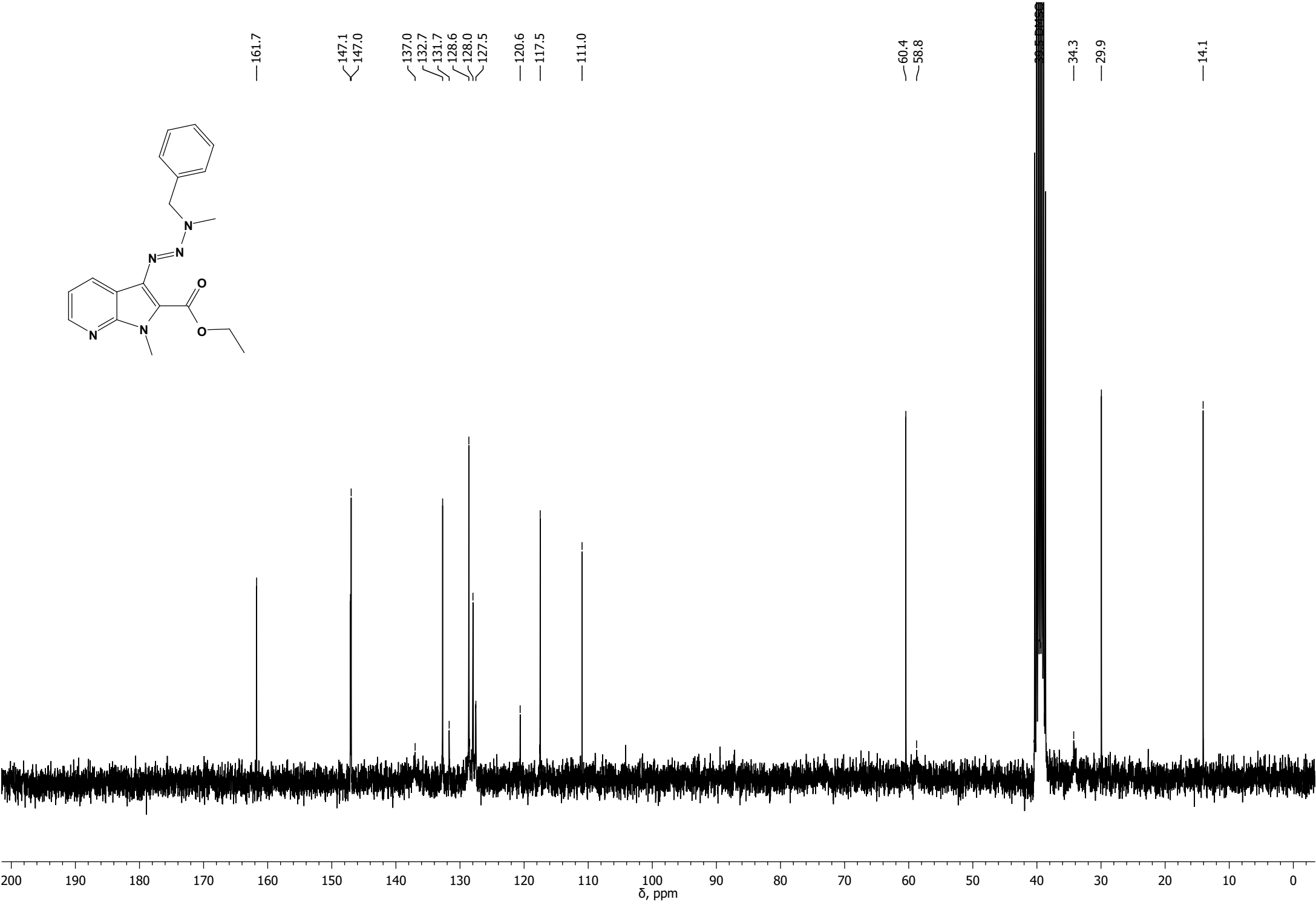

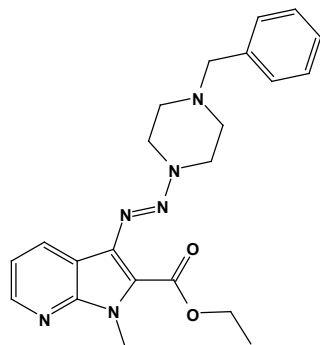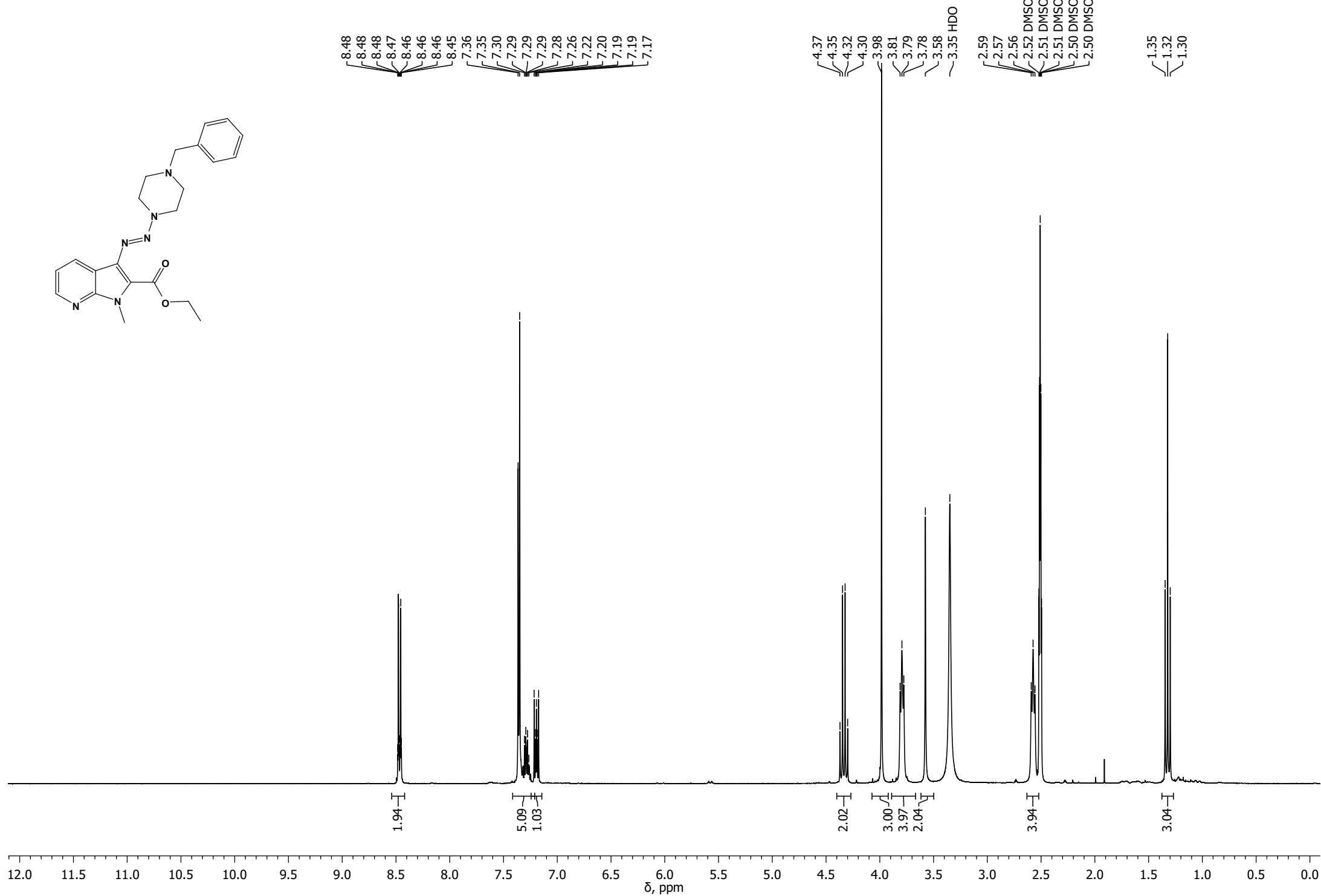

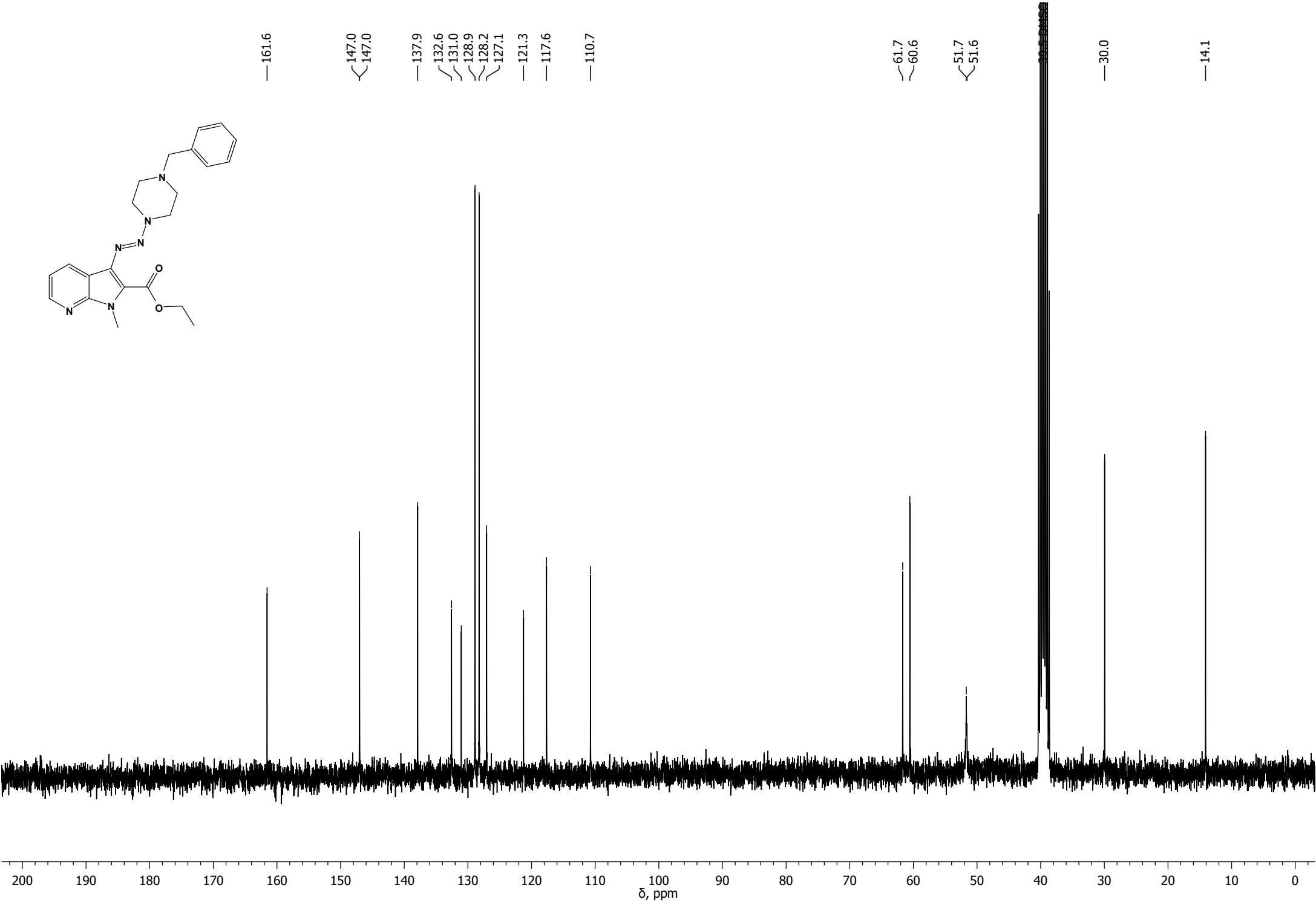

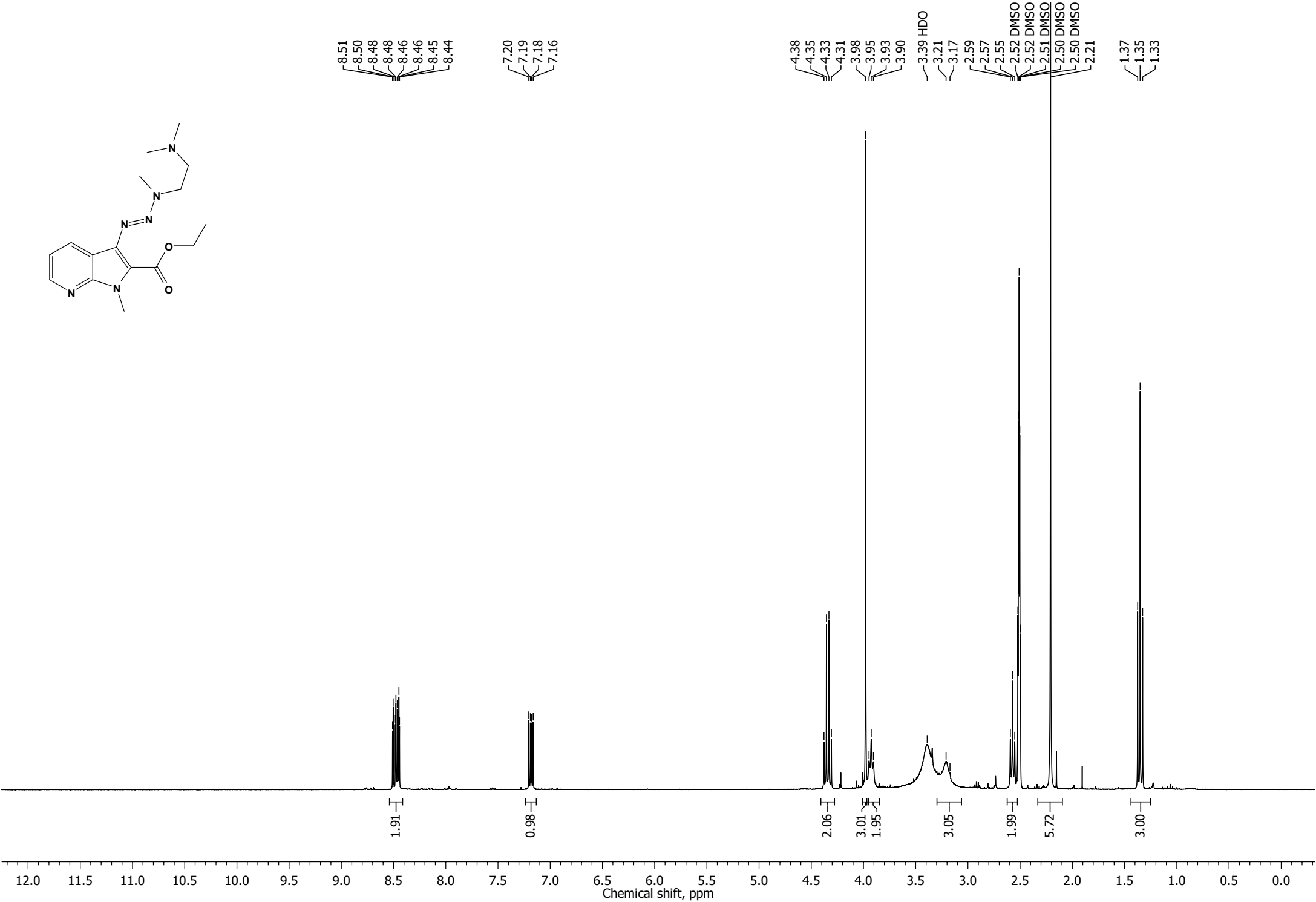

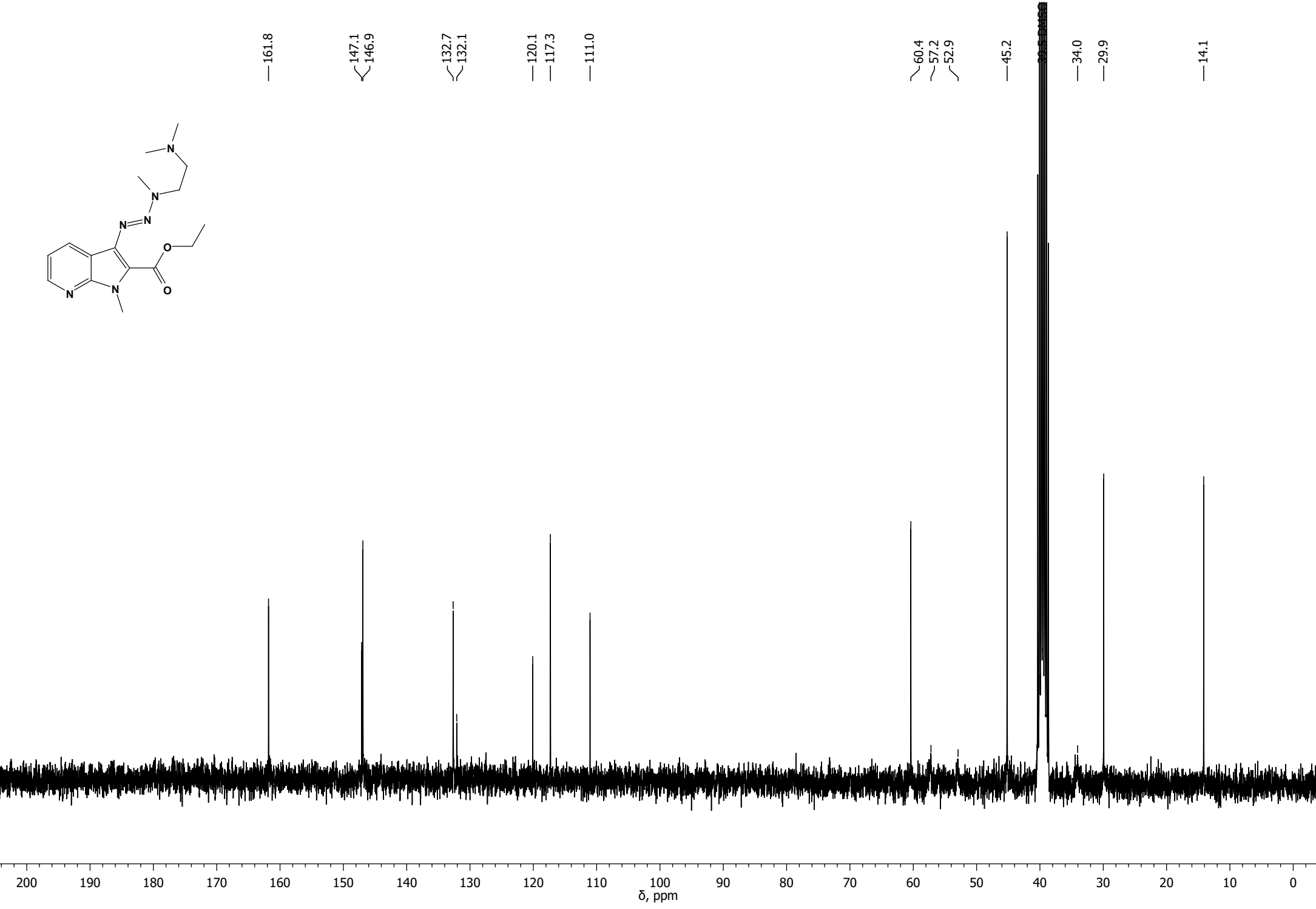

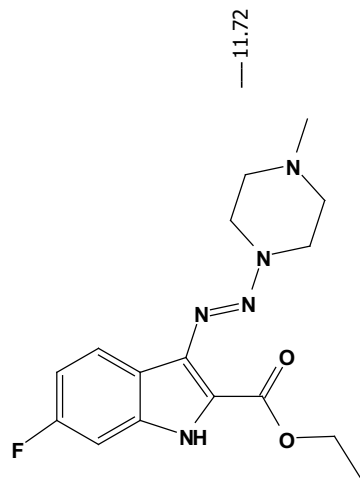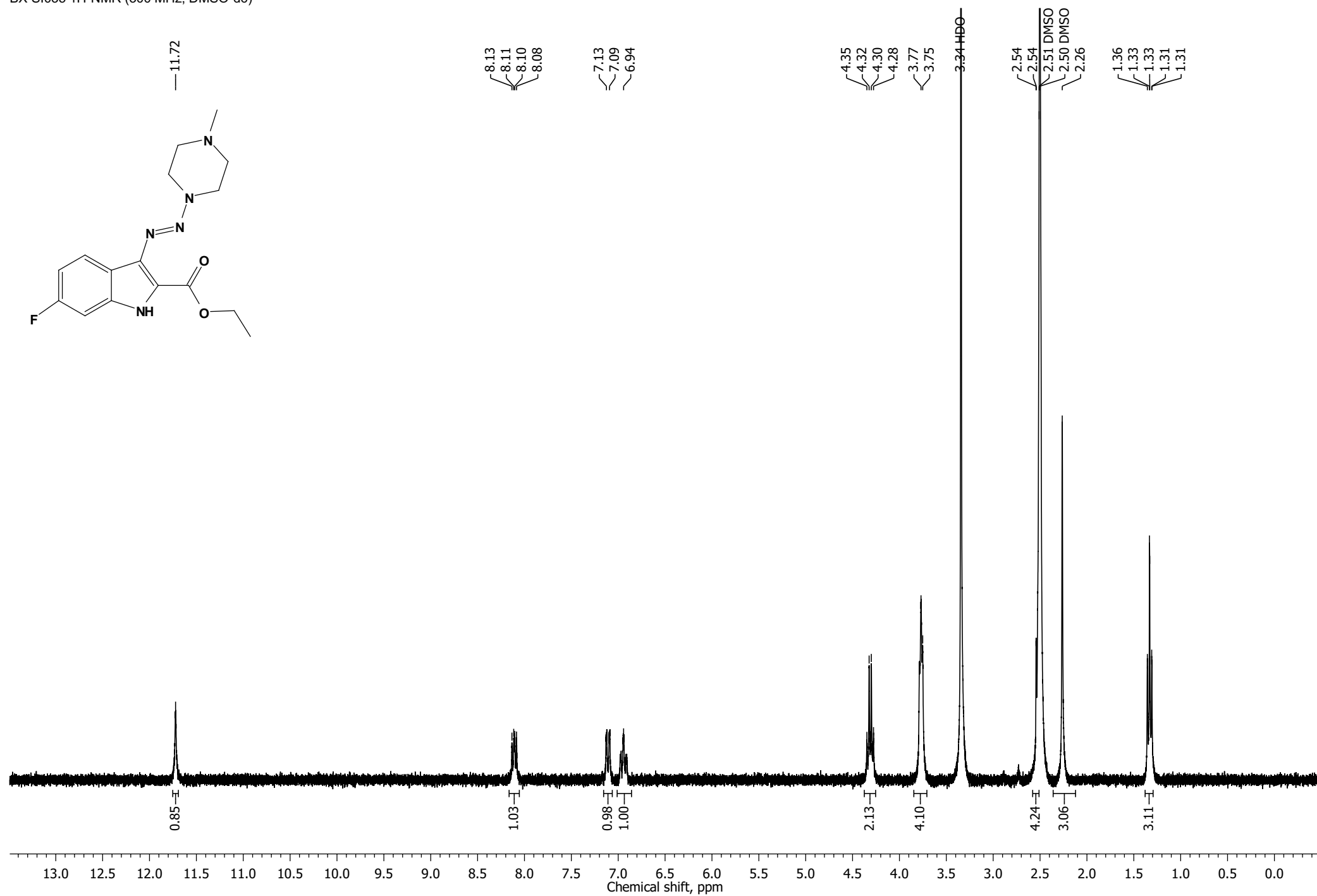

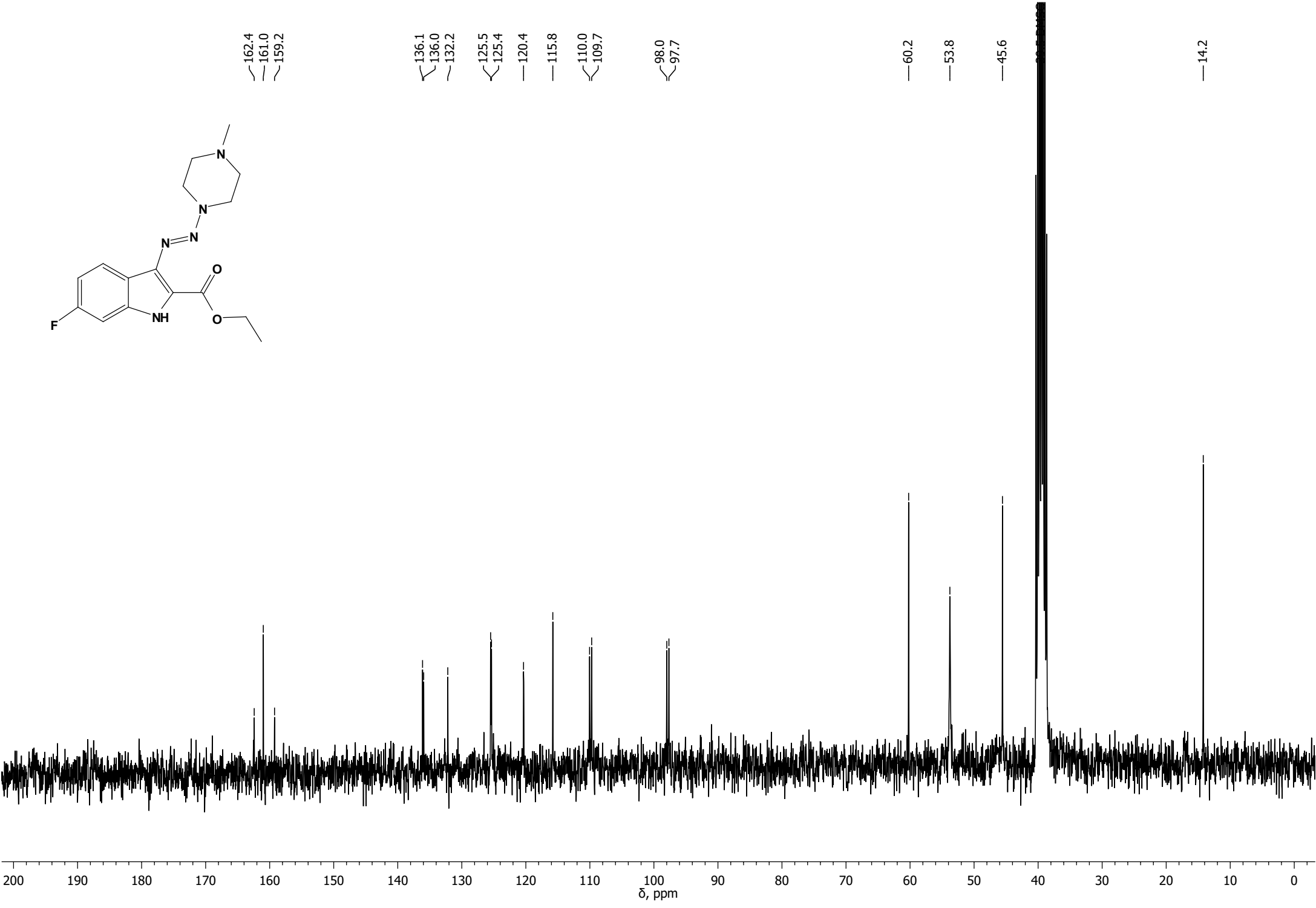

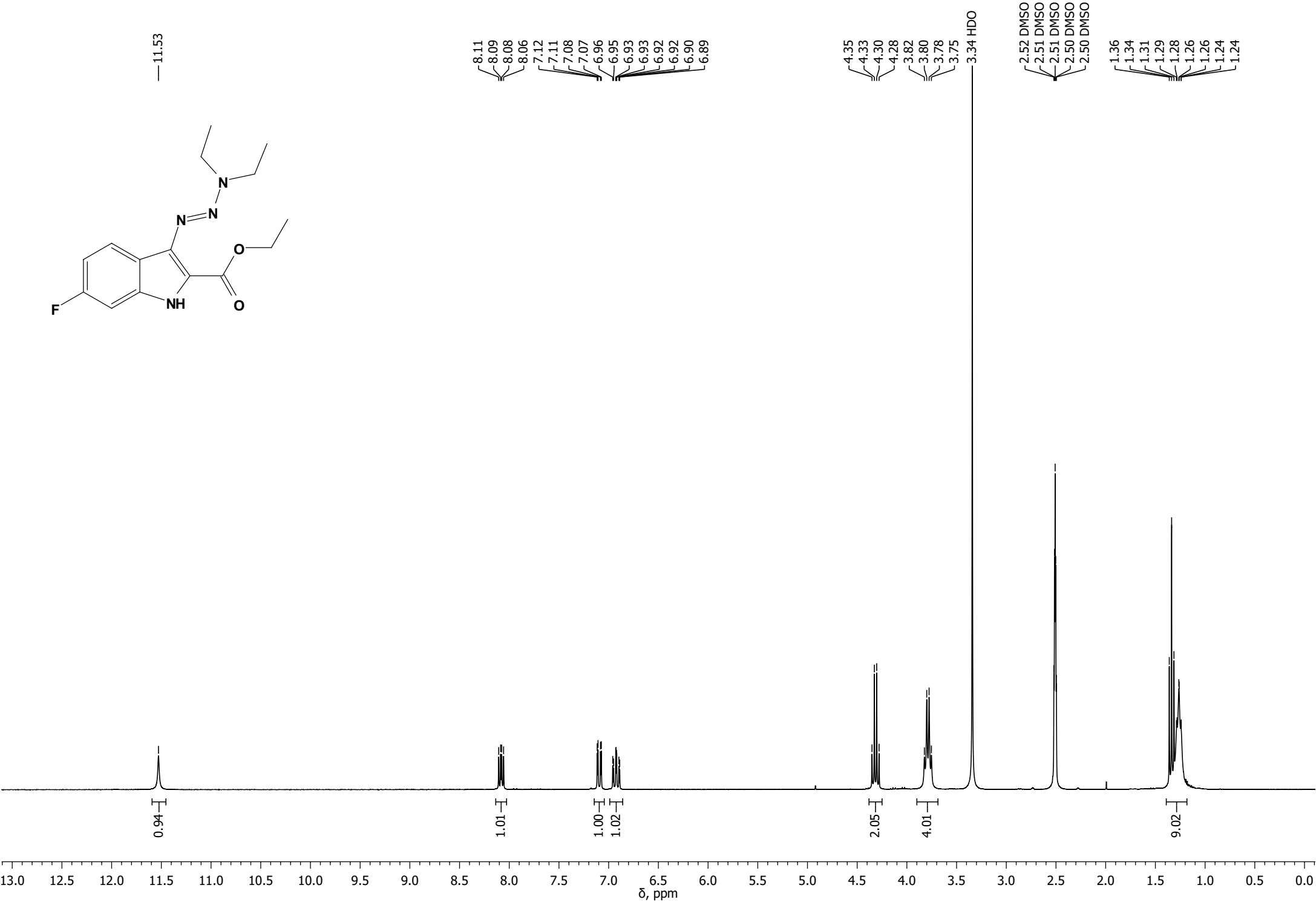

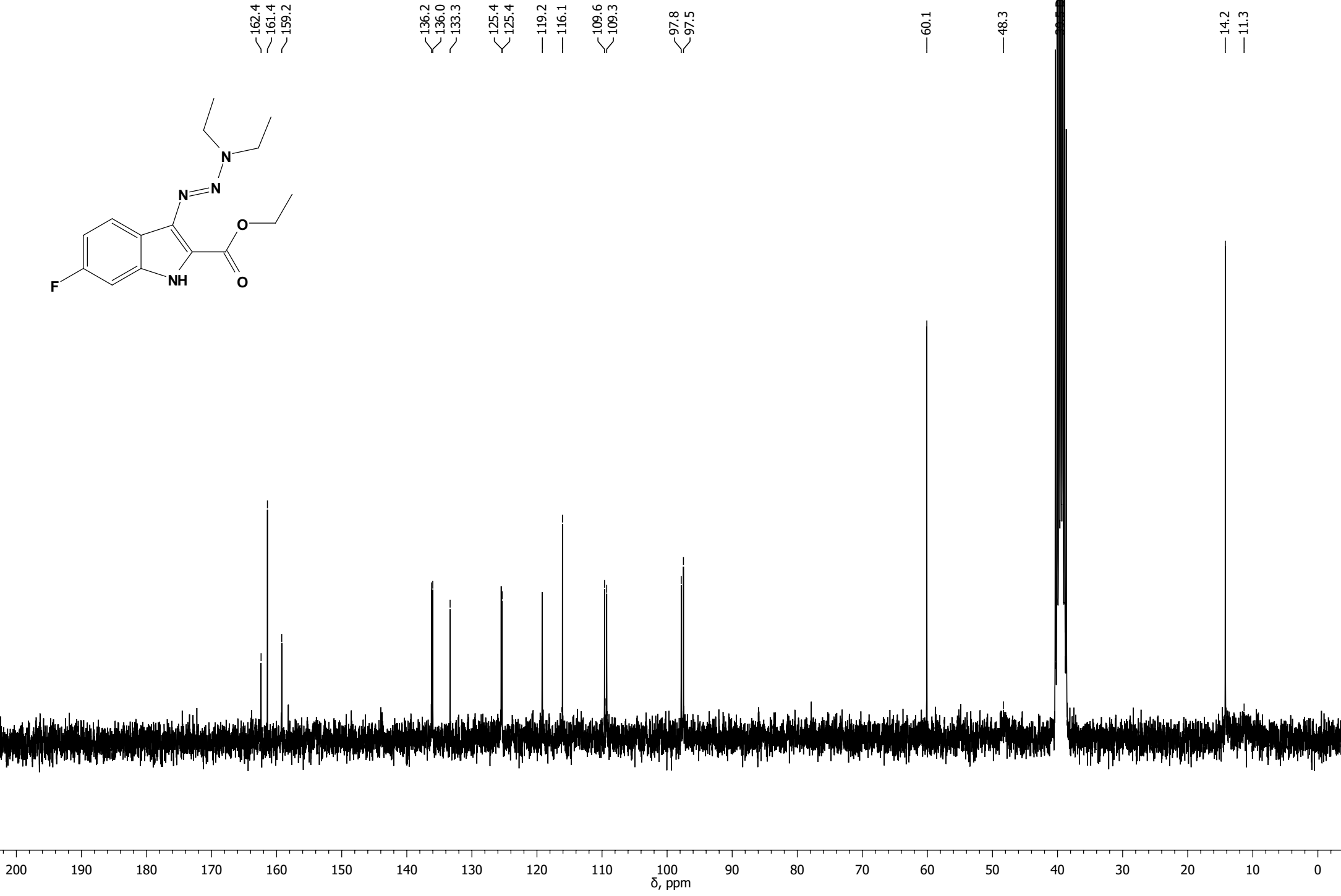

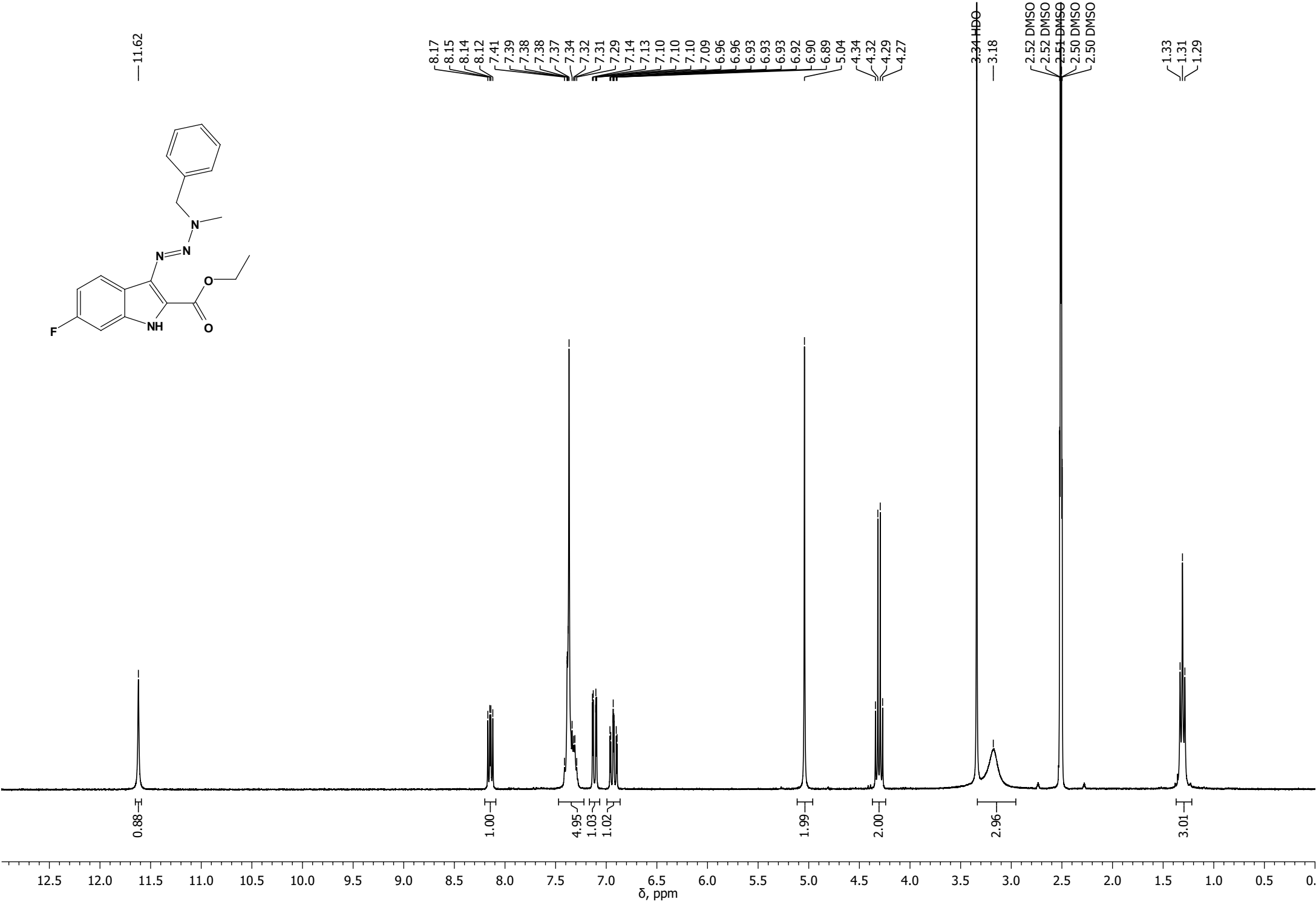

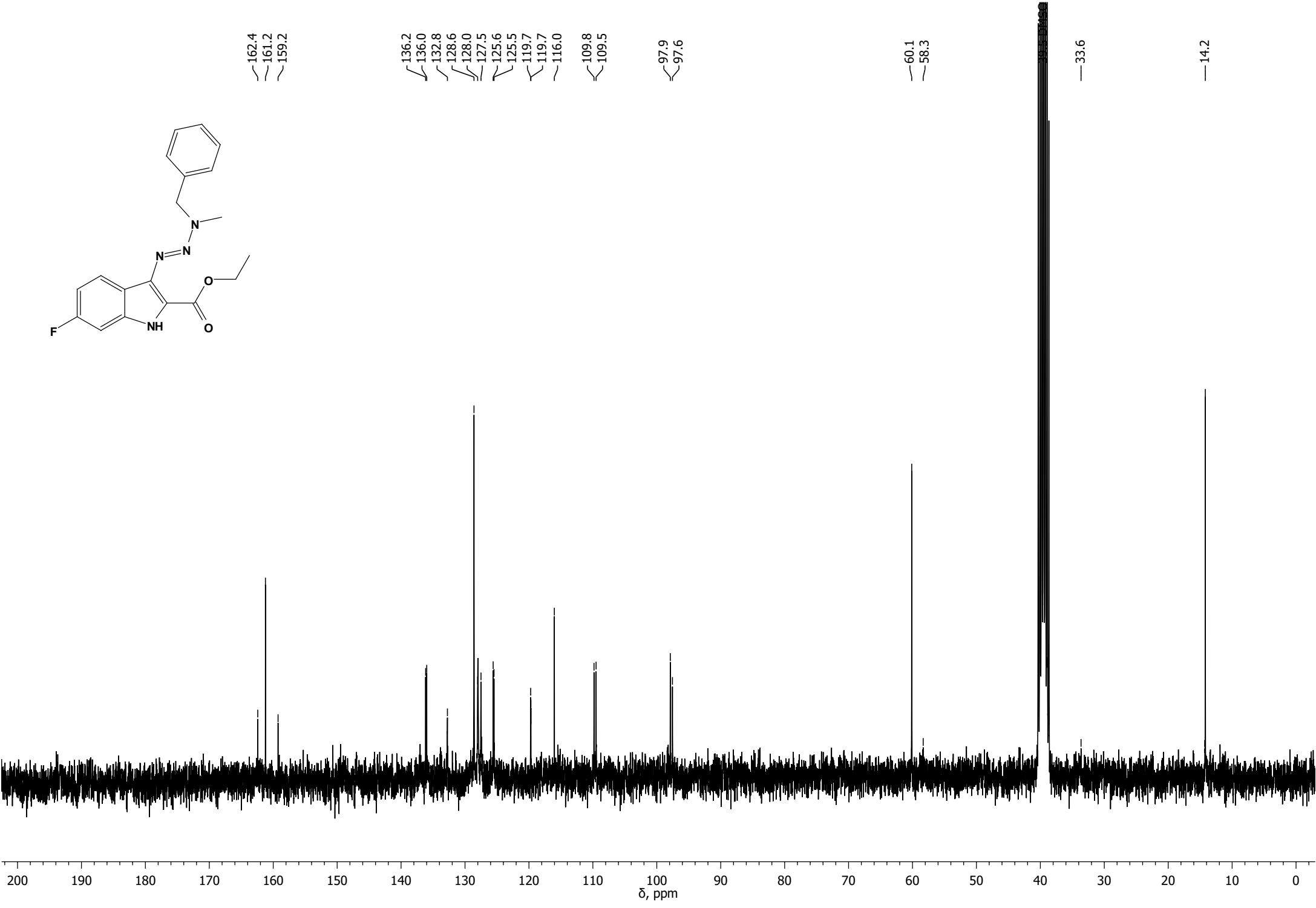

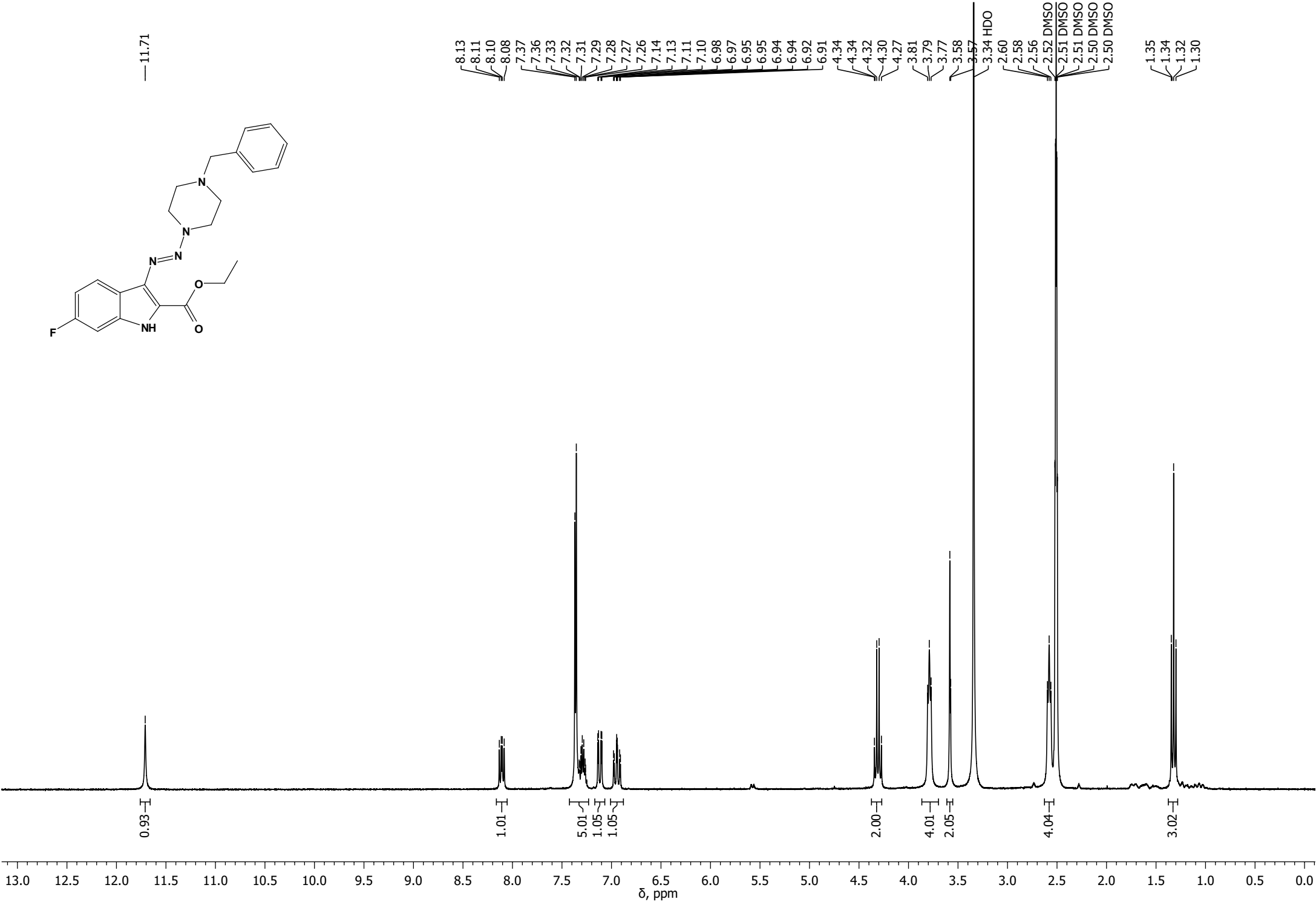

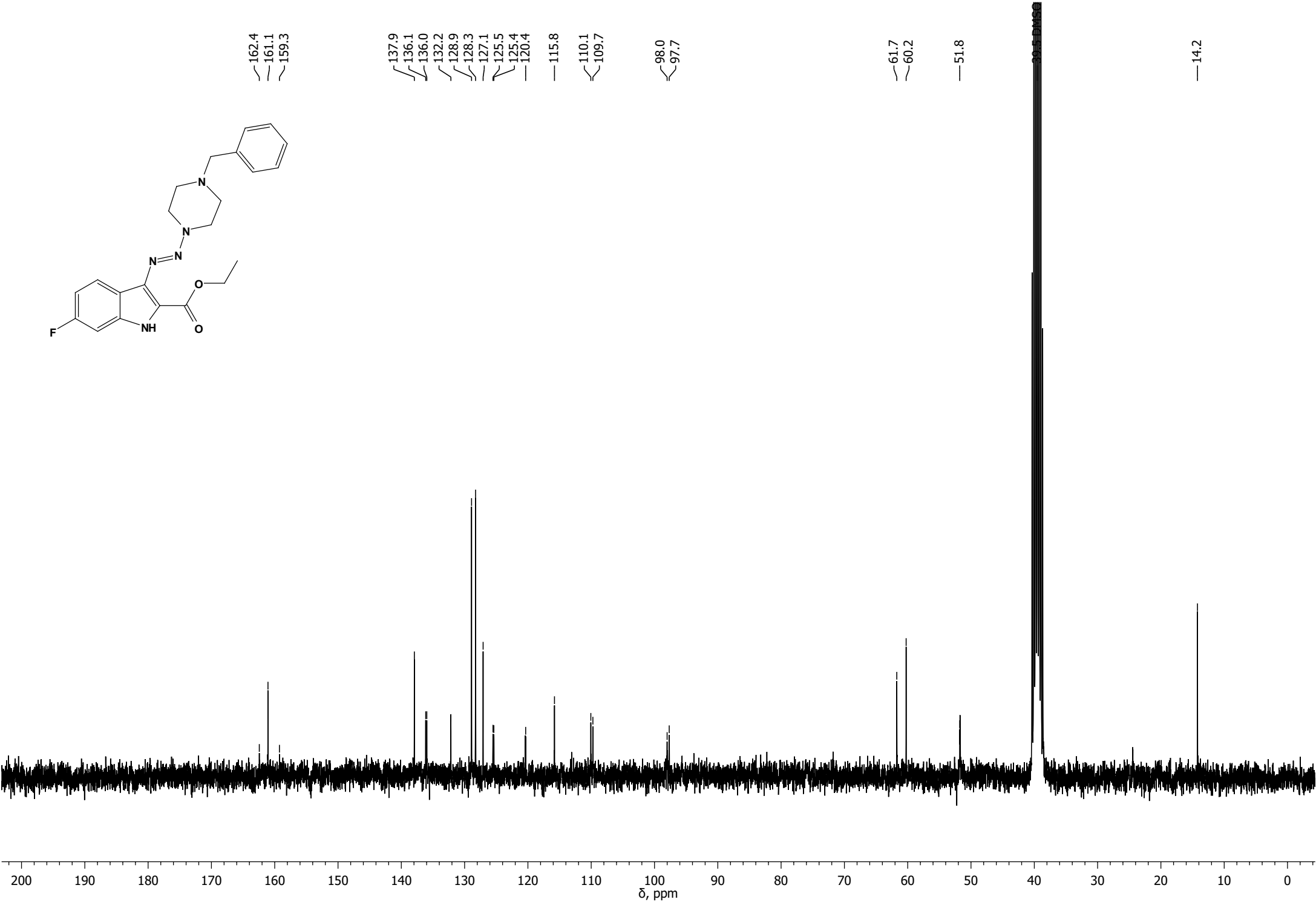

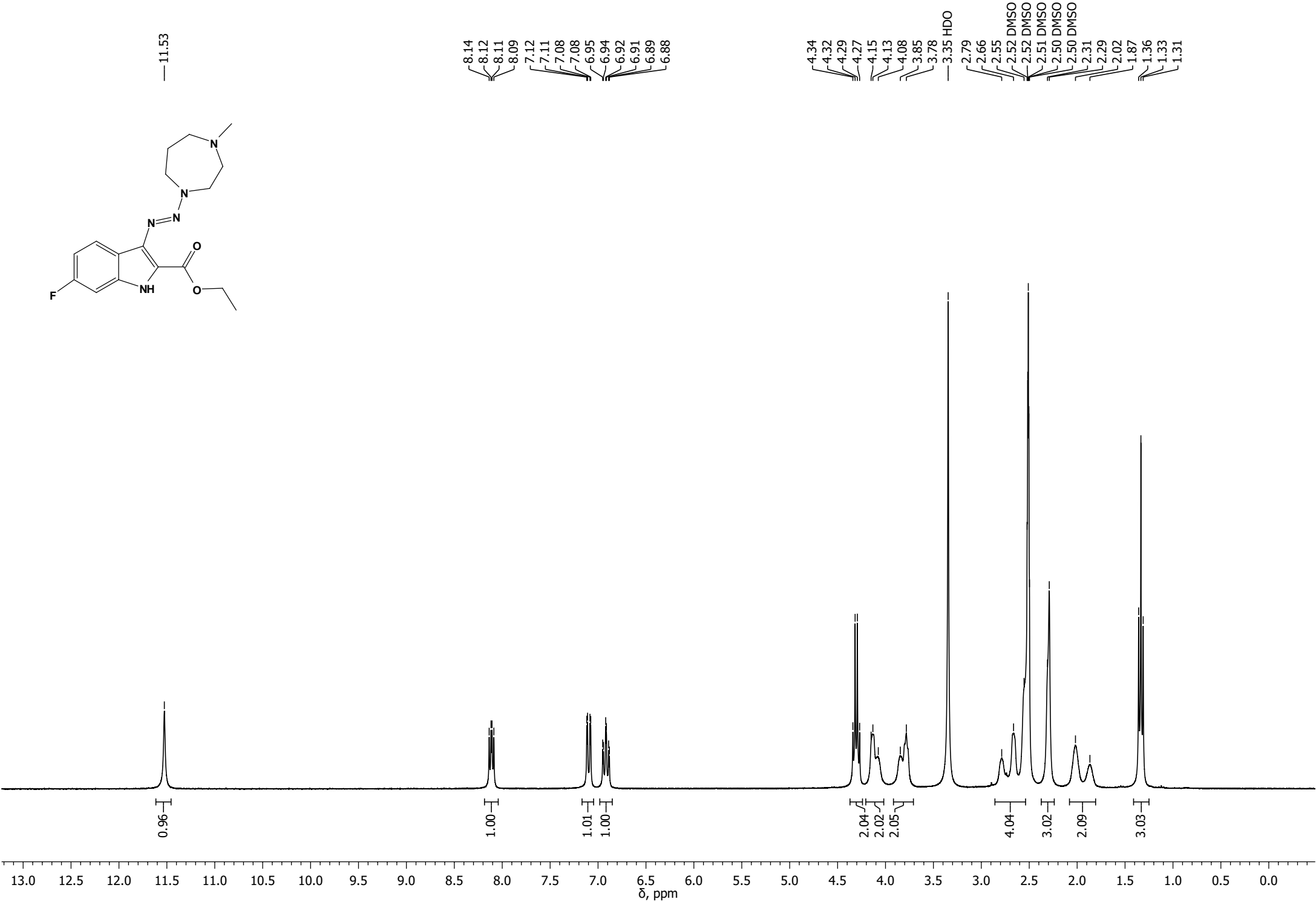

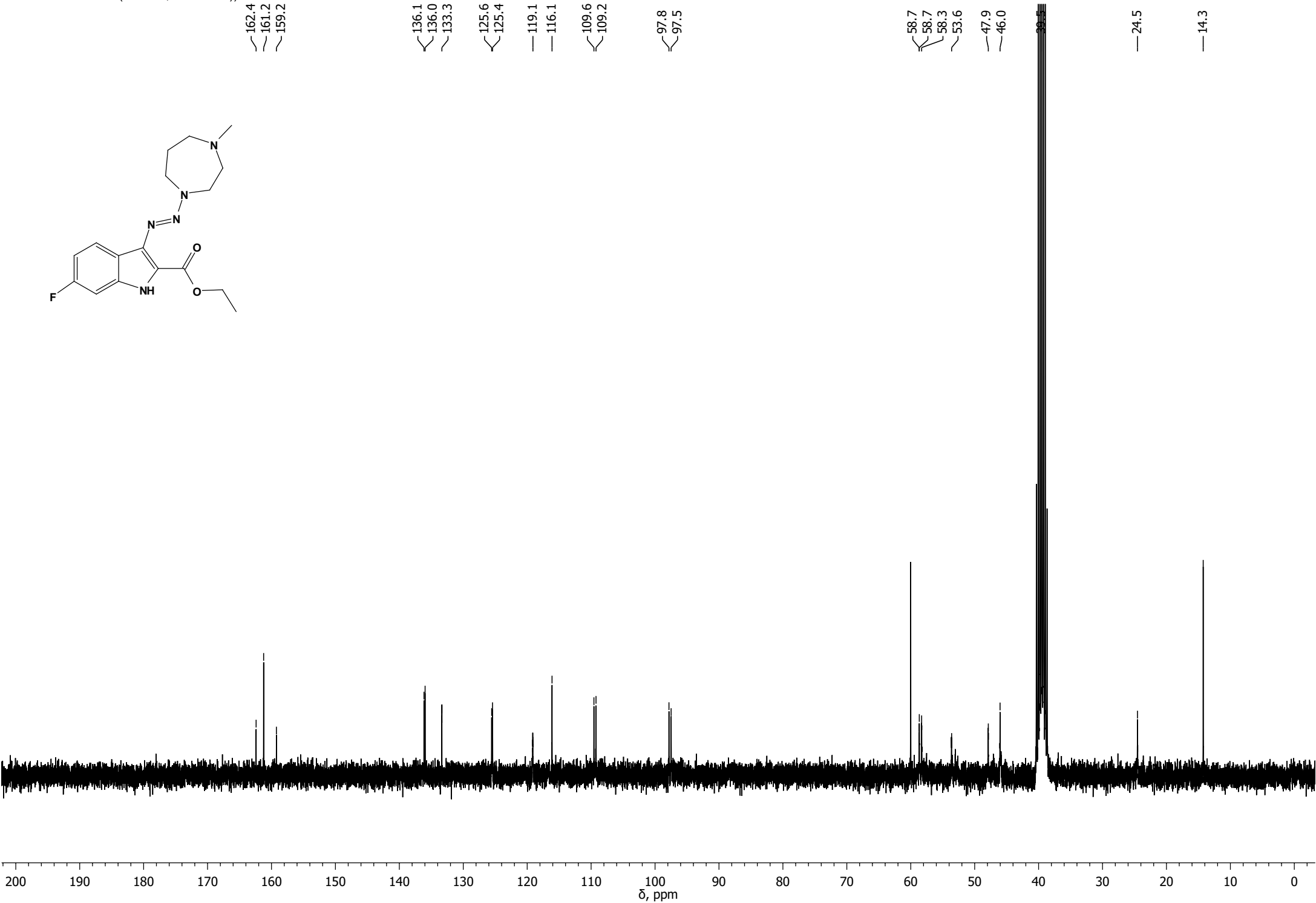

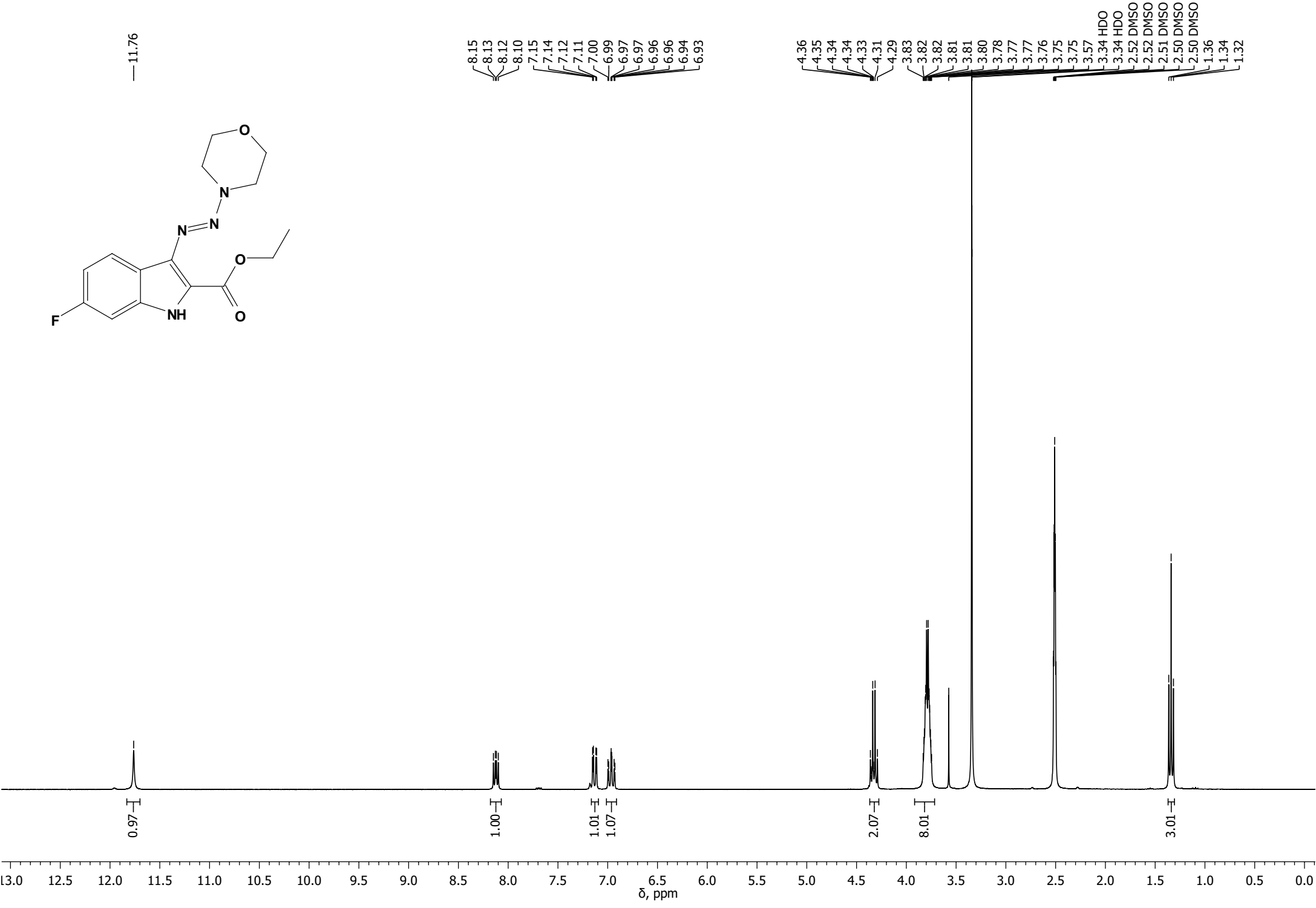

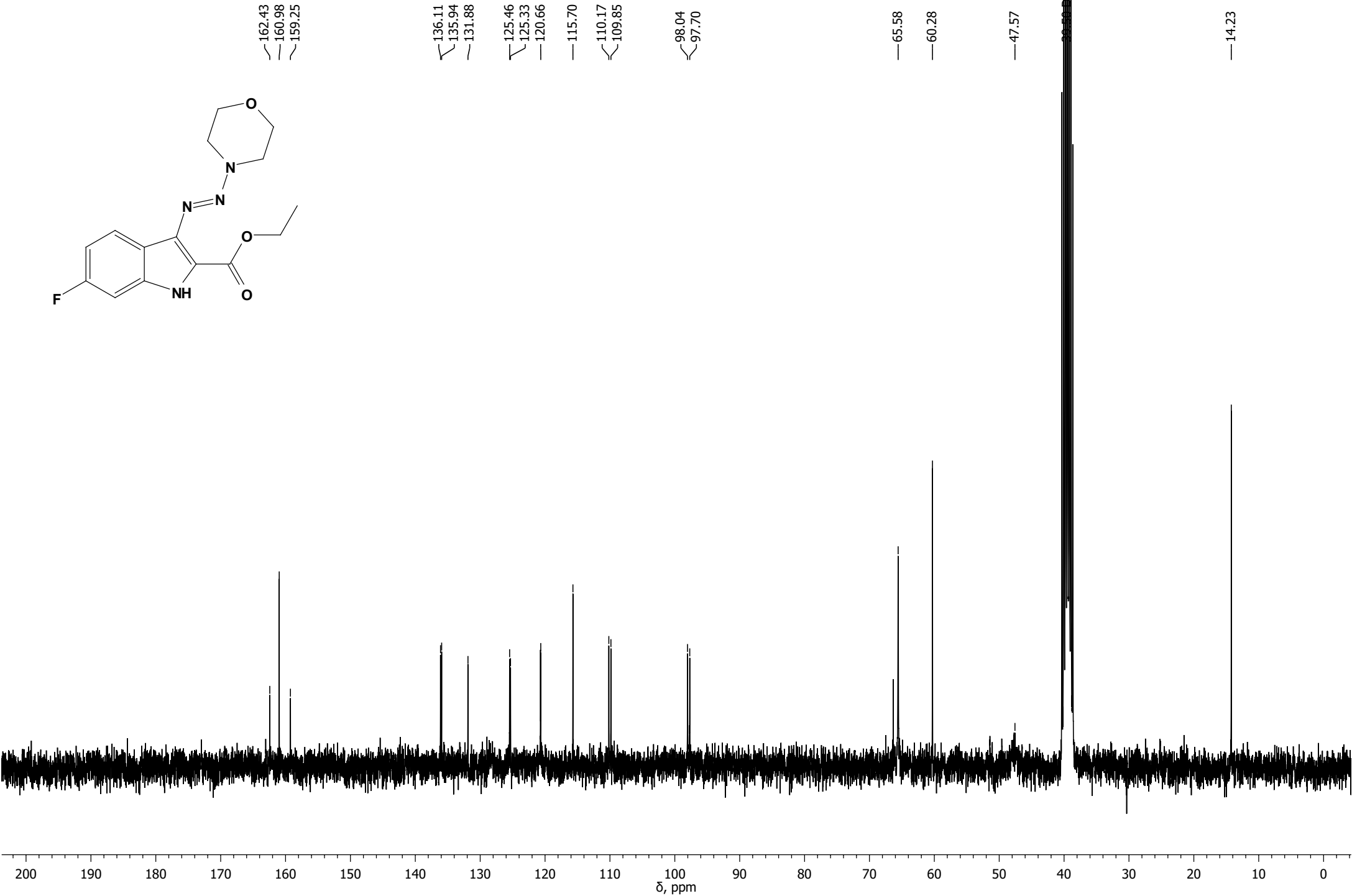

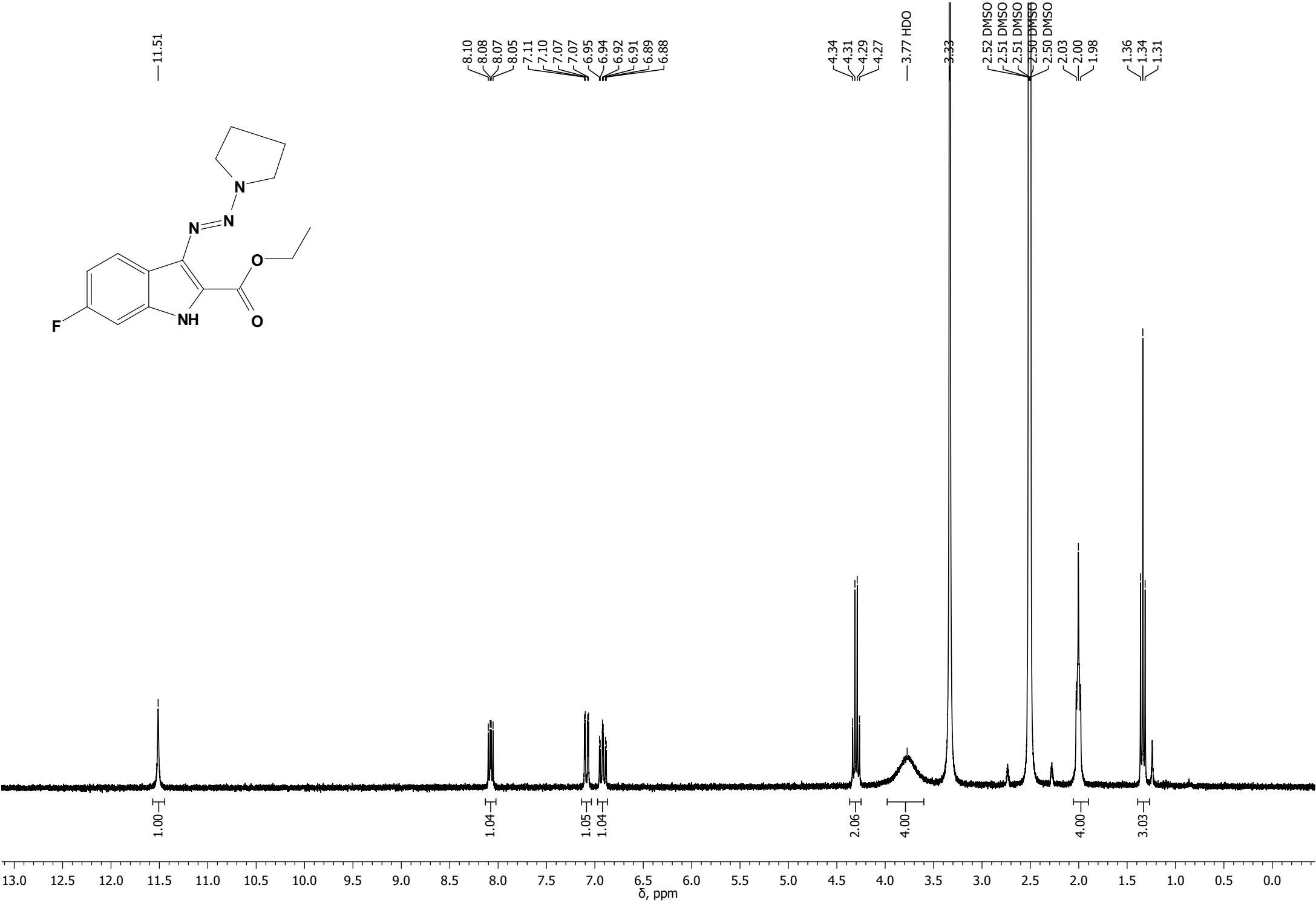

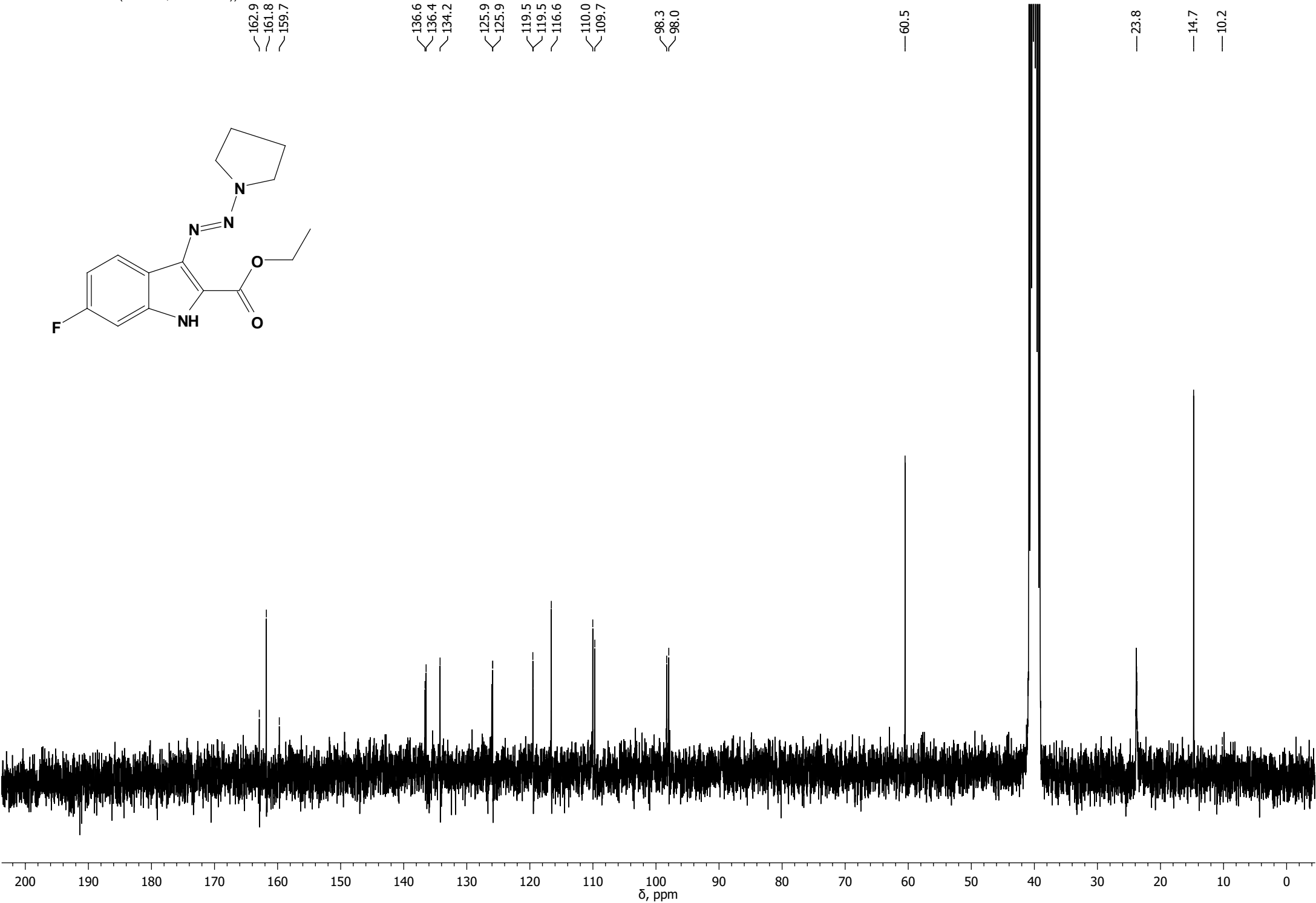

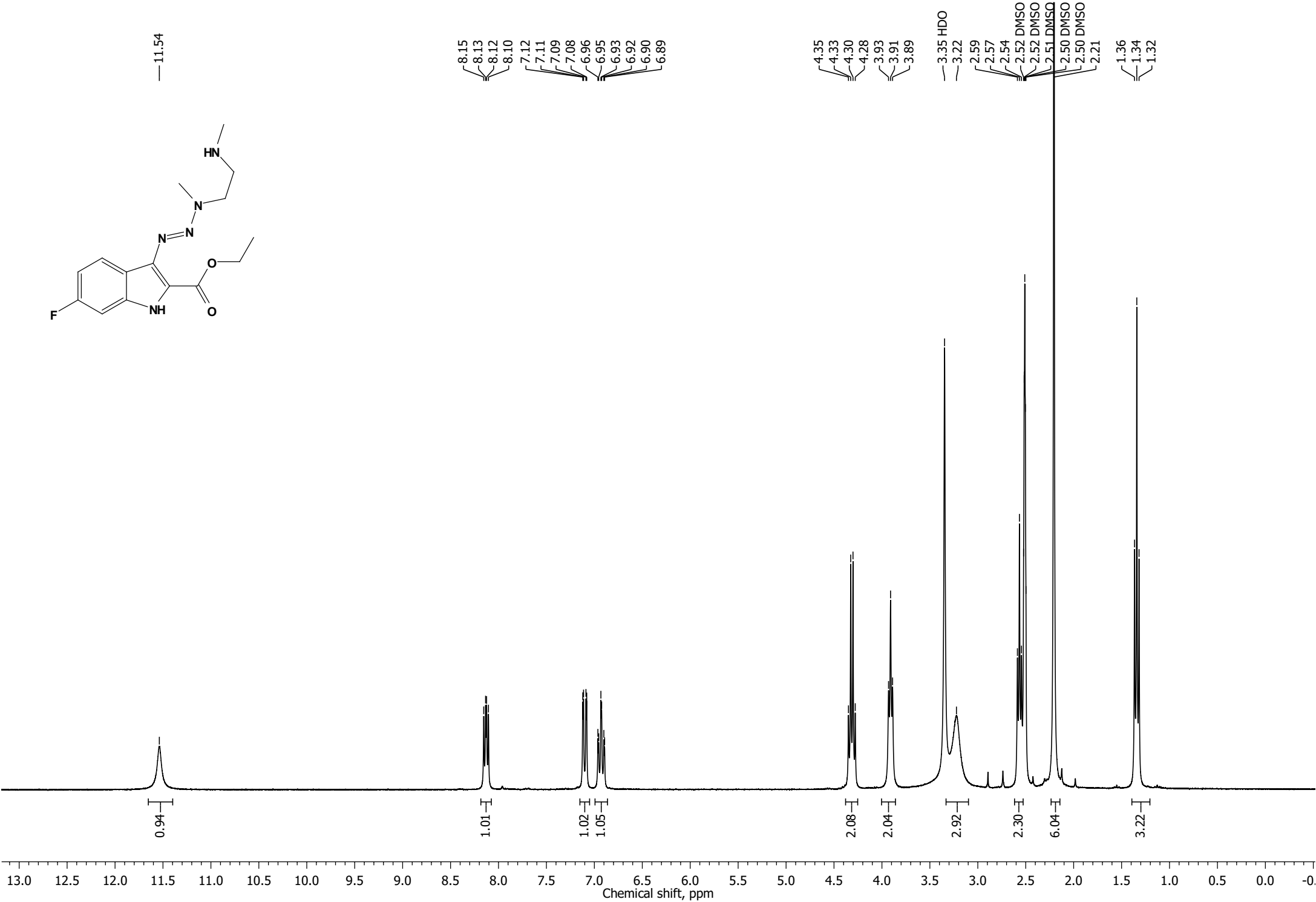

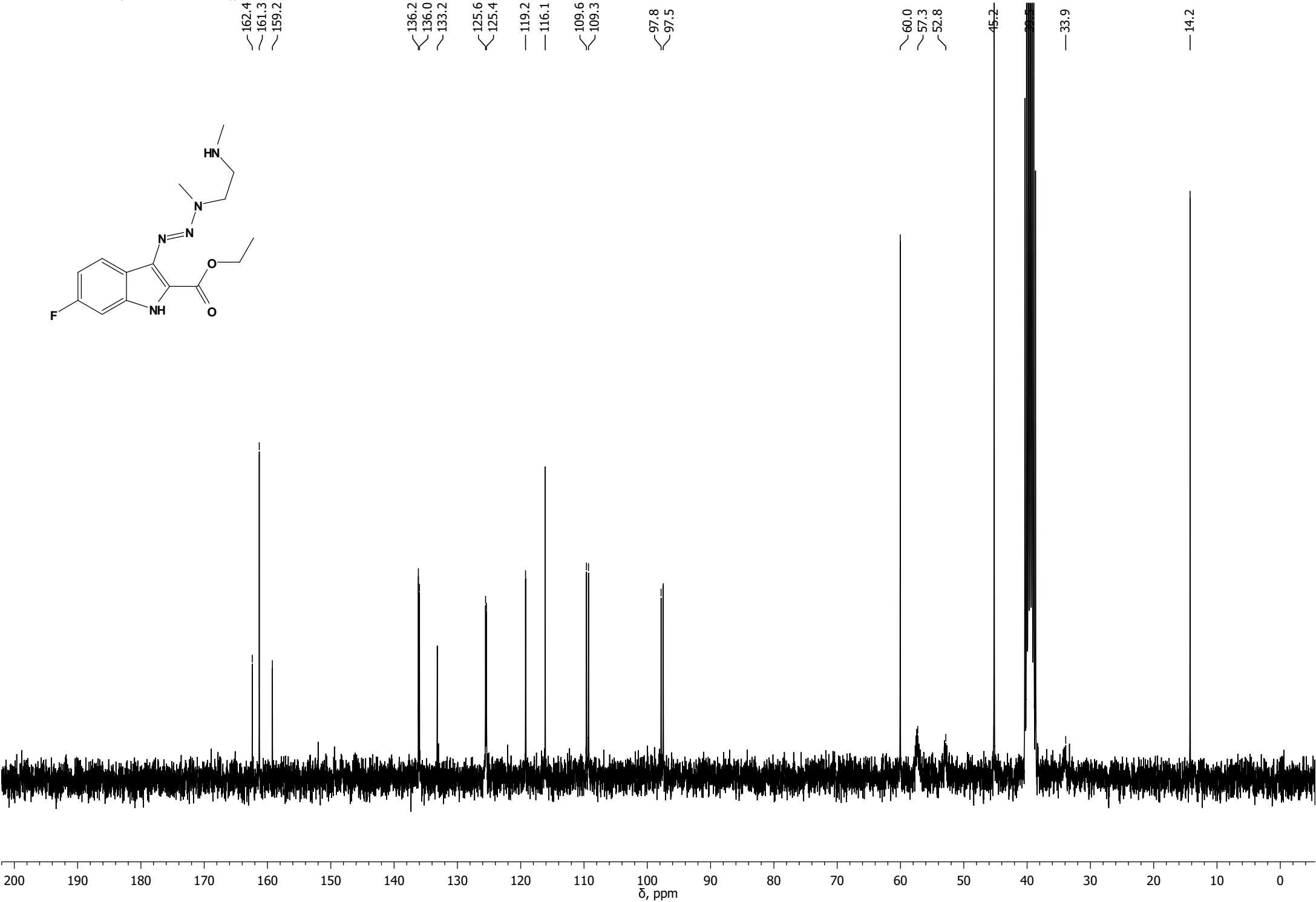

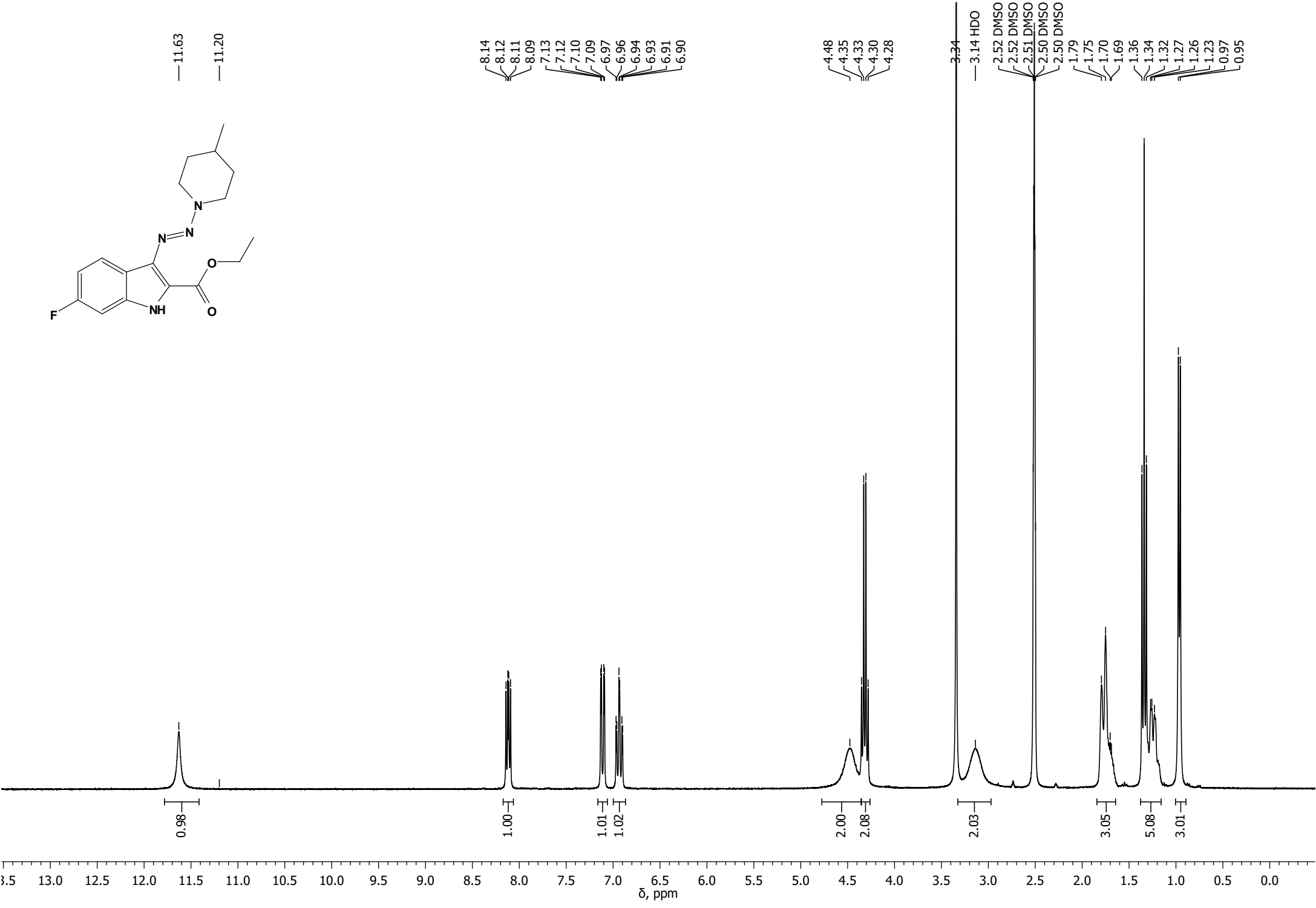

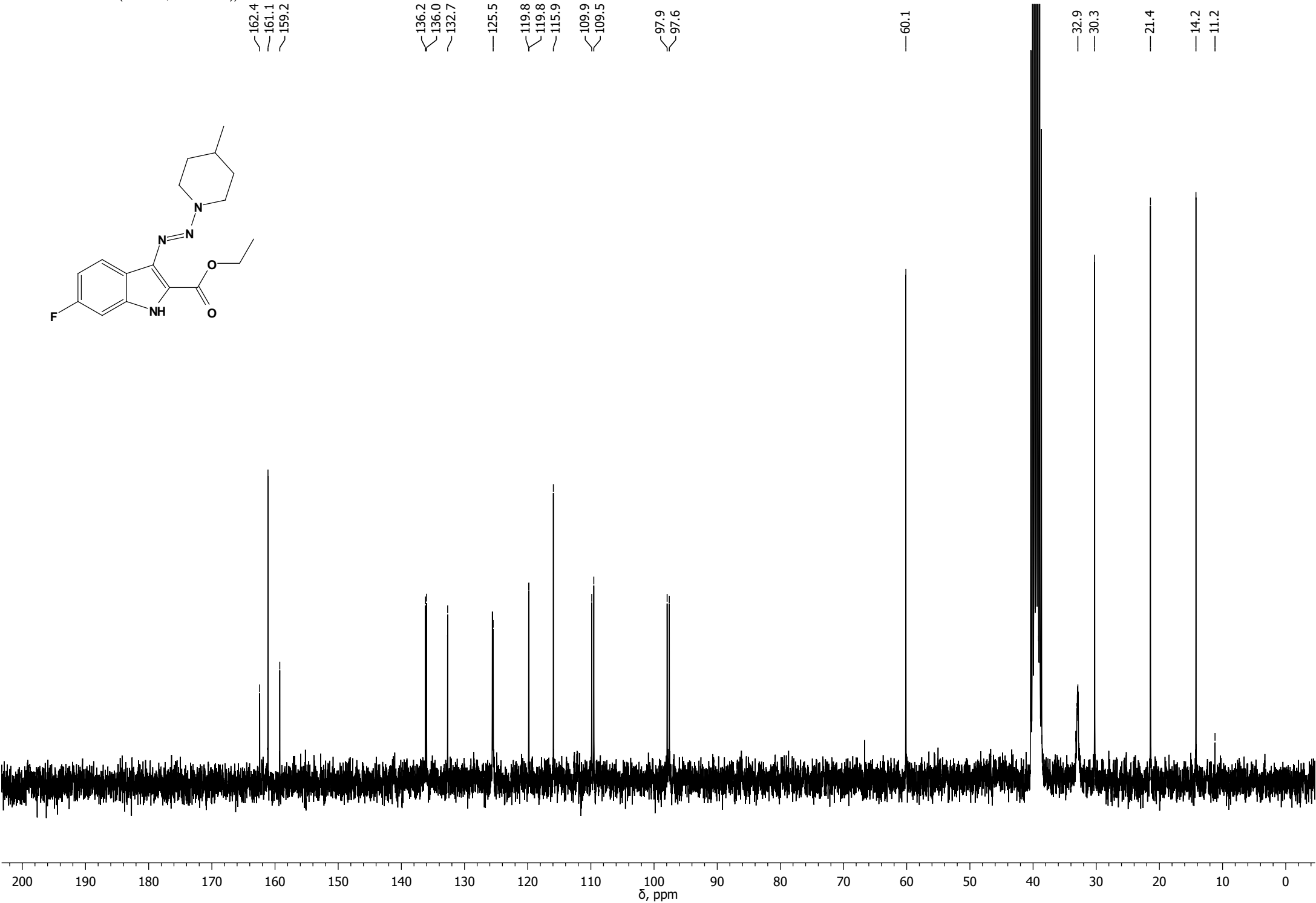

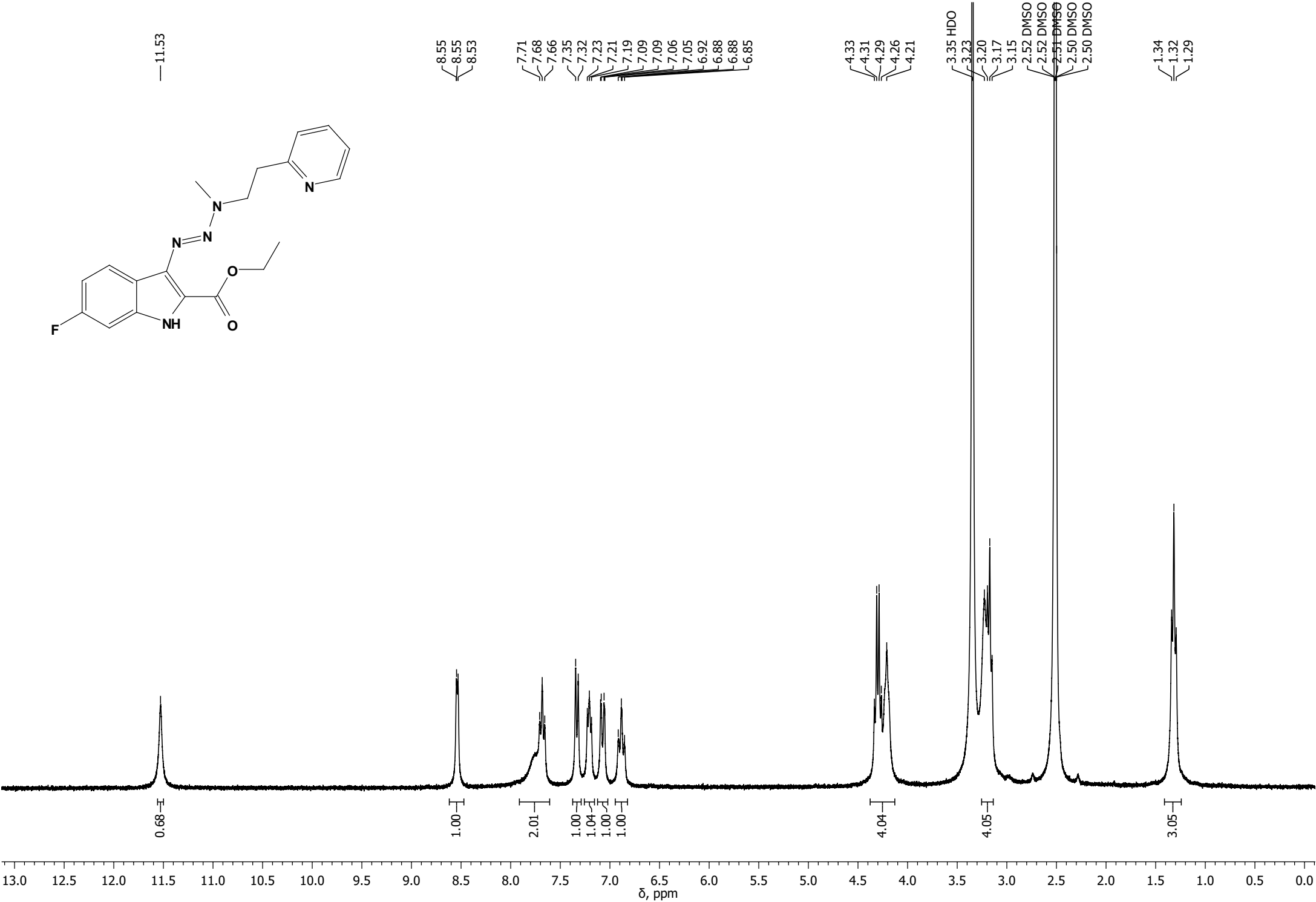

BX-SI048 <sup>13</sup>C-NMR (75 MHz, DMSO-d<sub>6</sub>)

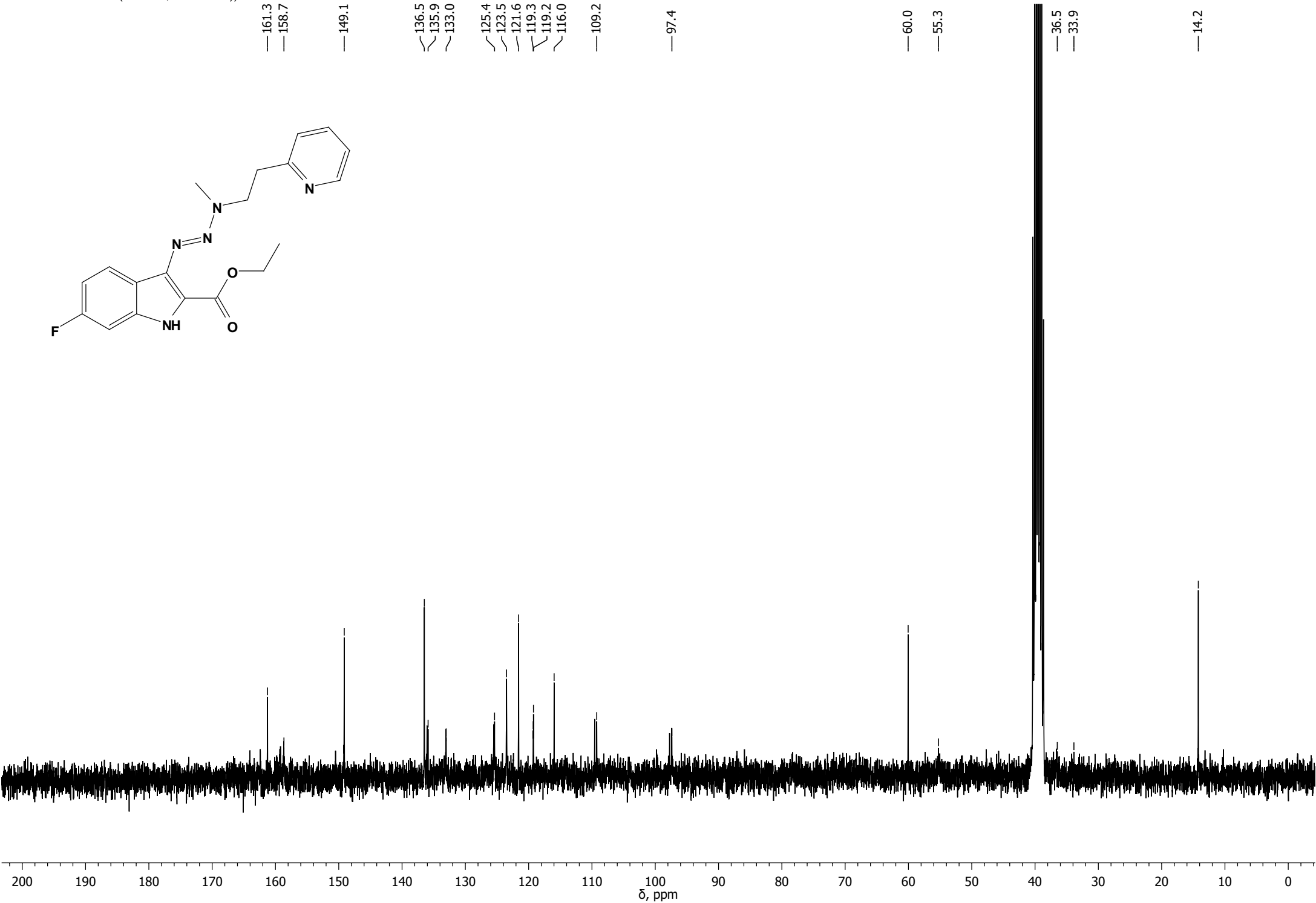

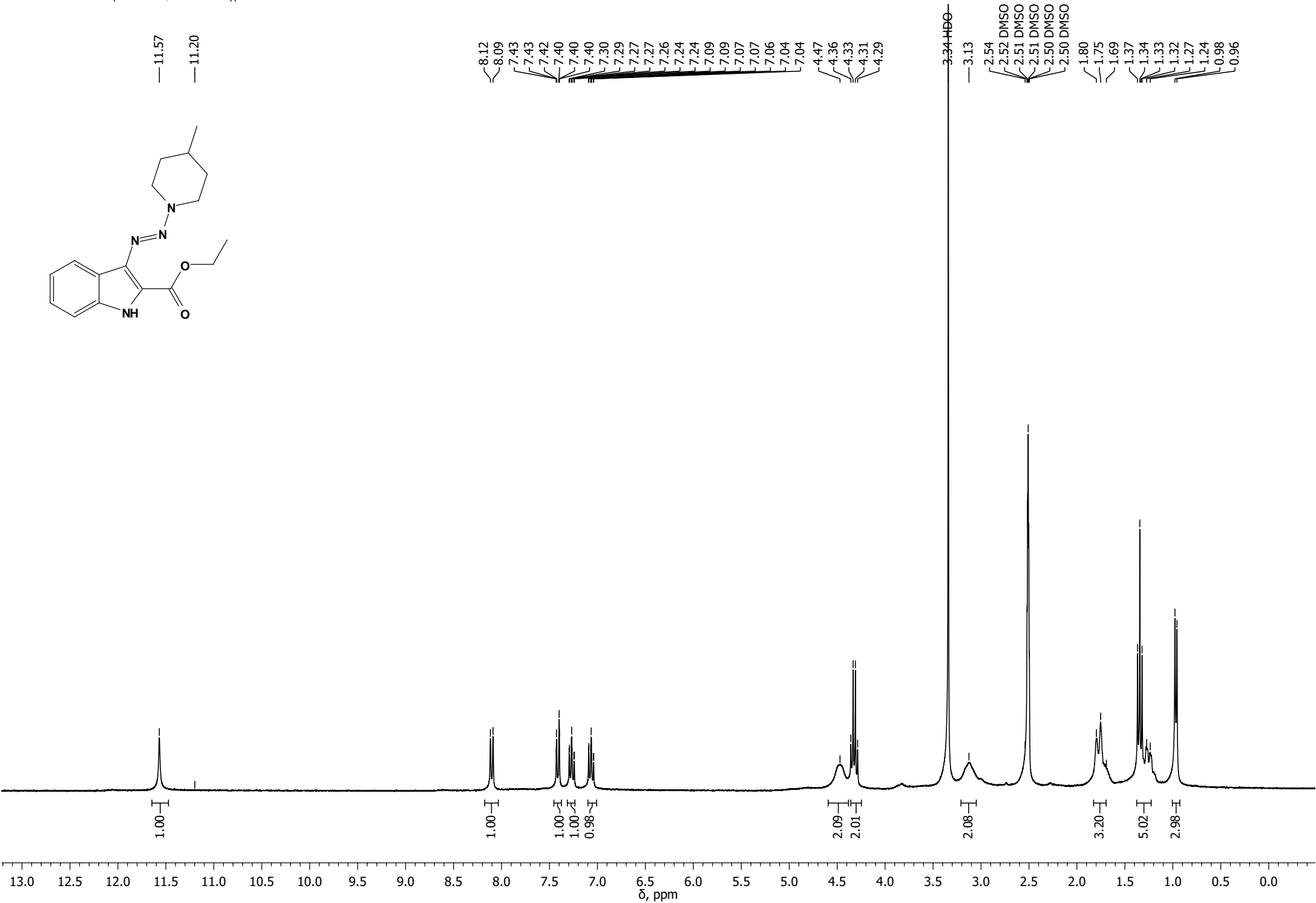

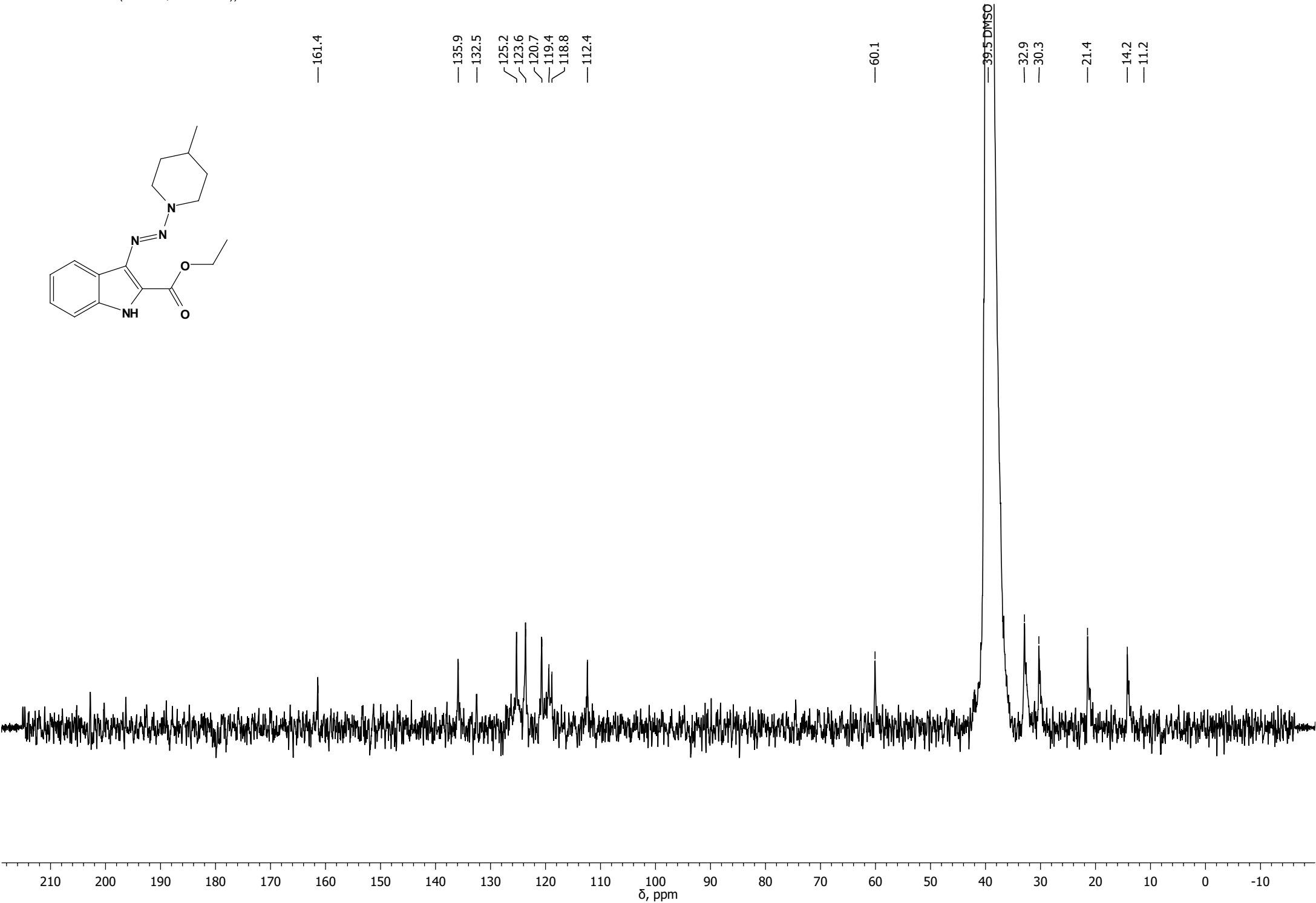

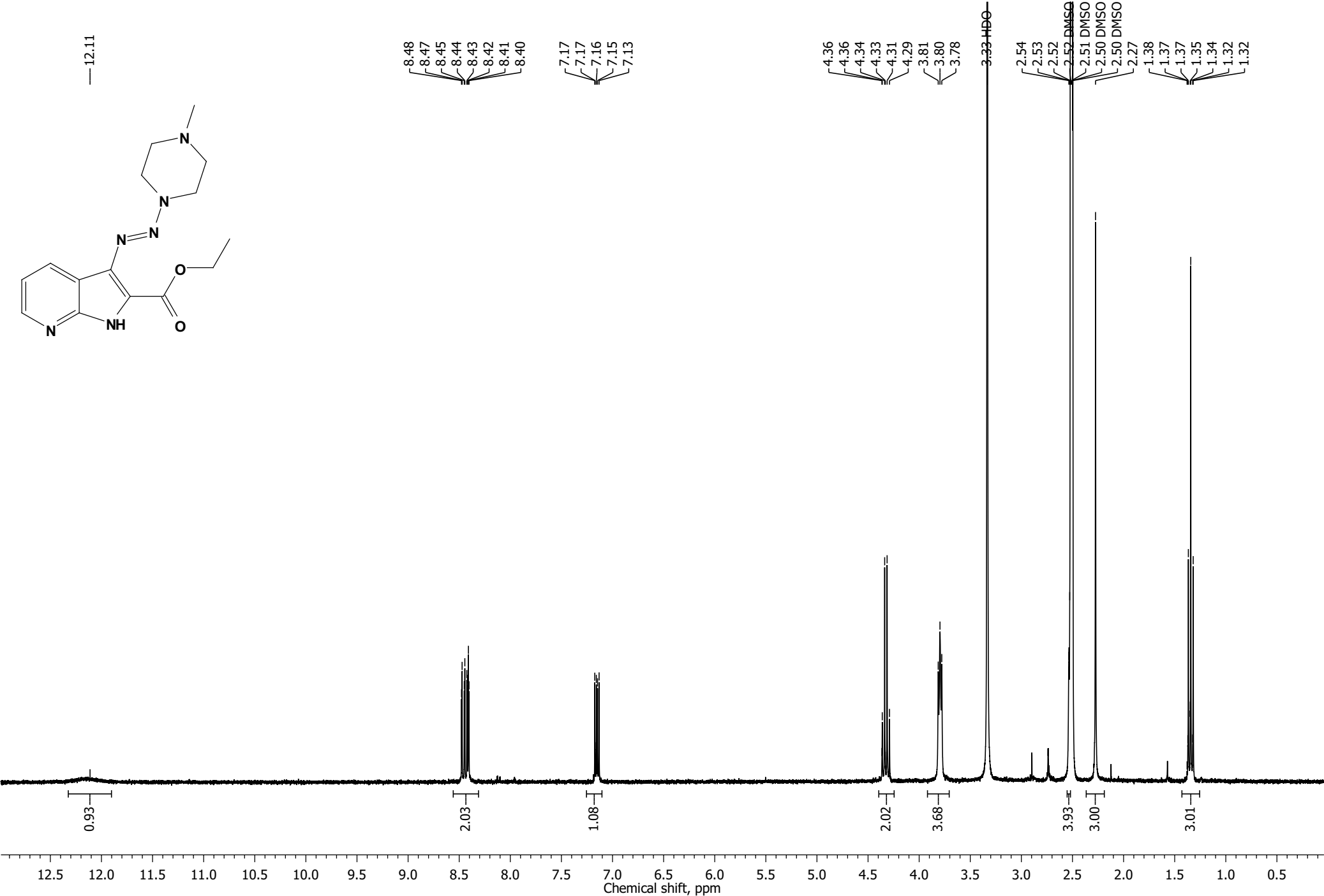

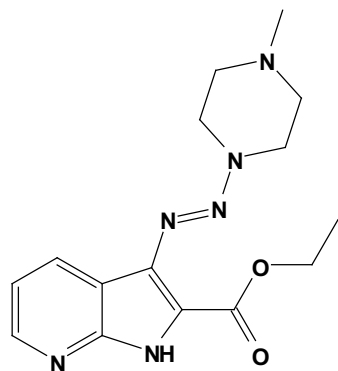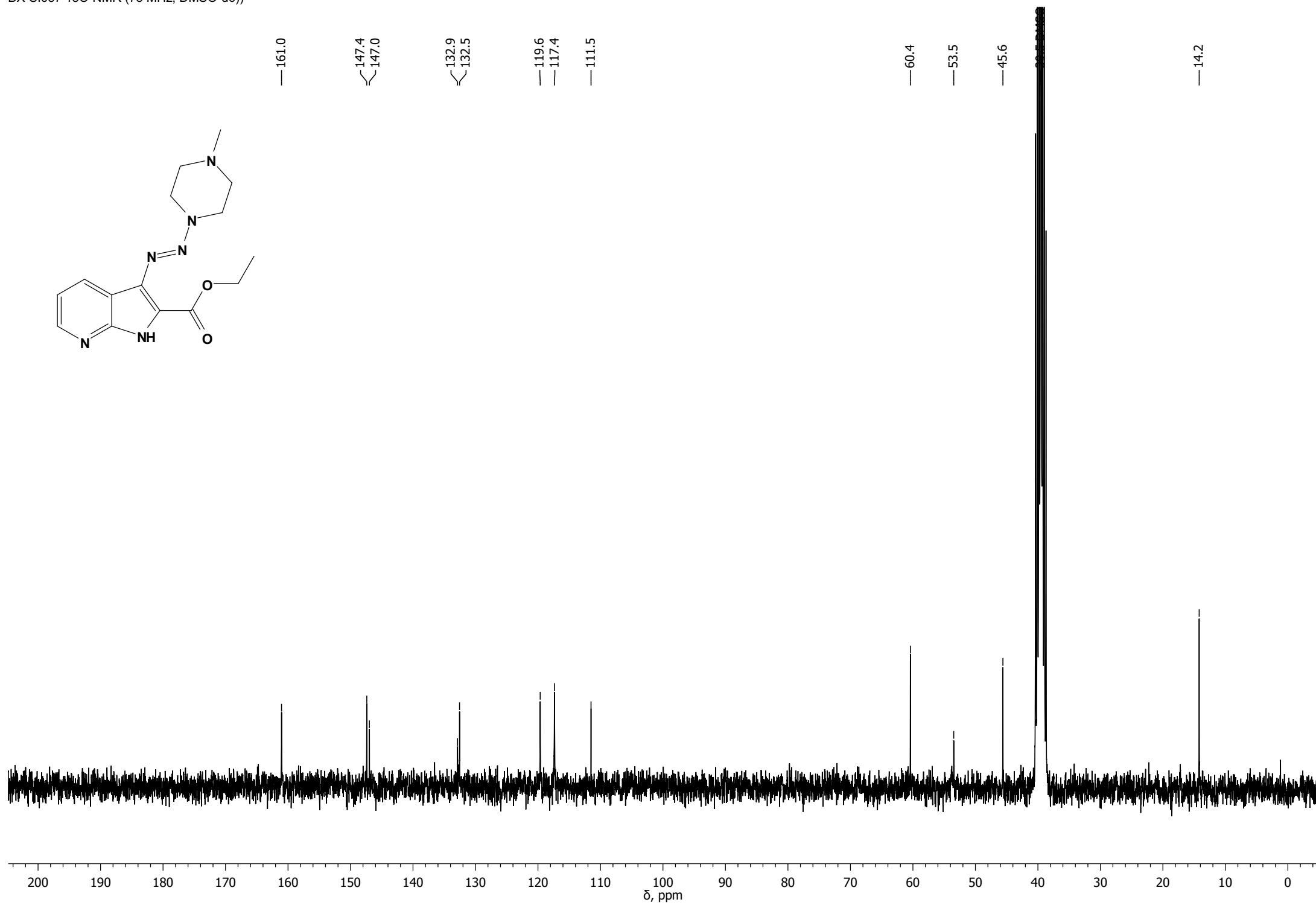

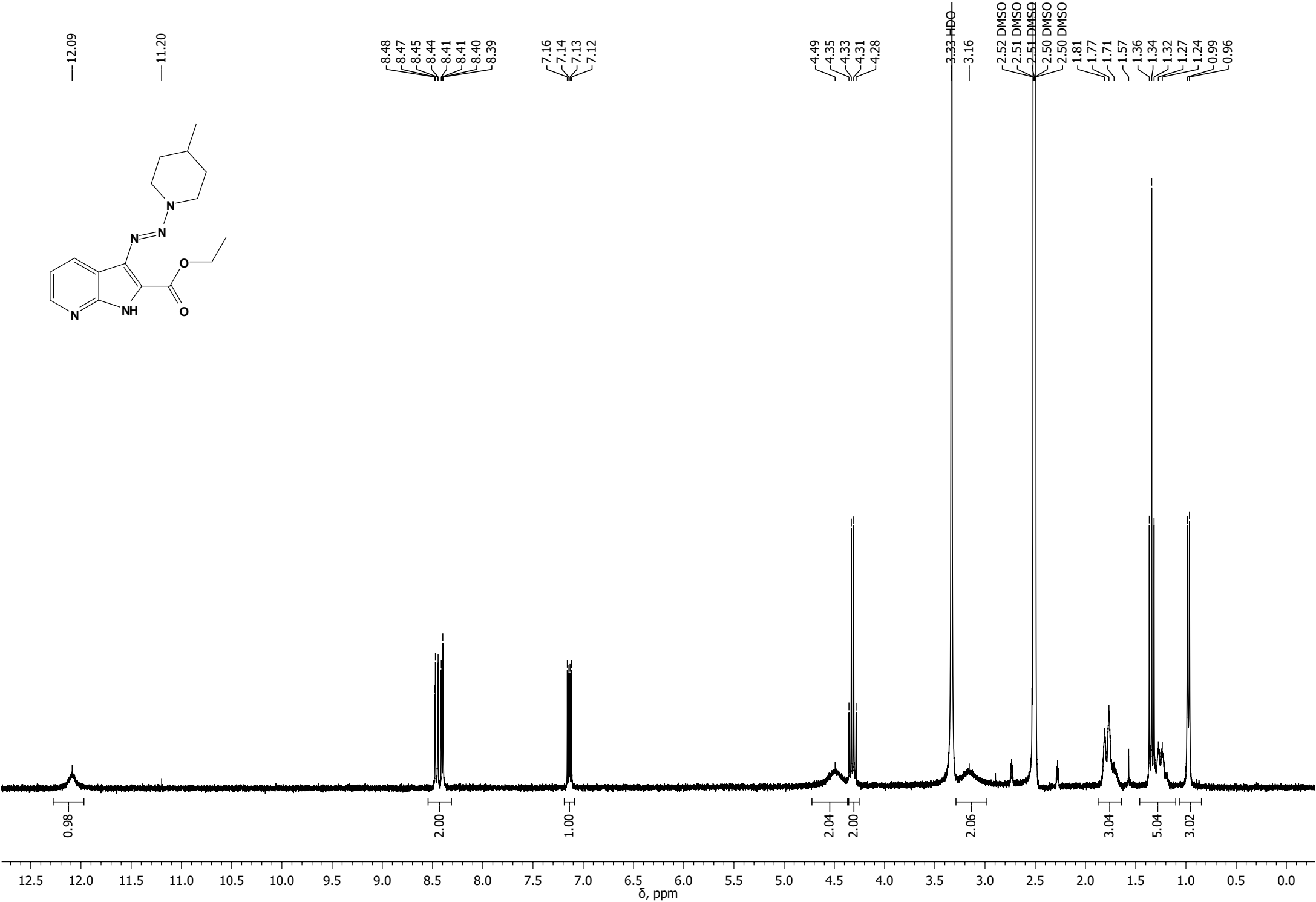

Supplement: Supplementary file 1 [file ijms-26-01870-s001.zip › Supplementary File S1.pdf]
